# Inhibition of YTHDF2-mediated CYLD mRNA degradation promotes neuronal ferroptosis and pain in Parkinson's disease through NOX4 deubiquitination

**DOI:** 10.1007/s10565-026-10204-0

**Published:** 2026-06-06

**Authors:** Fei Wang, Shuwei Bai, Yuanmei Pan, Zhongjiao Lu, Xianguo Jiang

**Affiliations:** 1 Department of Neurology, Renji Hospital, School of Medicine, Shanghai Jiao Tong University, Shanghai, 200127 P. R. China; 2 Department of Neurology, Shanghai Clinical Research and Trial Center, Shanghai, 201210 P. R. China

**Keywords:** YTHDF2, CYLD, NOX4, Parkinson’s disease, Ubiquitination, M^6^A

## Abstract

**Background:**

Parkinson’s disease (PD) is featured by progressive neurodegeneration linked to iron-dependent ferroptosis, yet the functions of m^6^A RNA-binding proteins and deubiquitinating enzymes in this process remain poorly understood. This work investigates the functional interplay between the m^6^A reader YTHDF2 and deubiquitinase CYLD in PD-associated ferroptosis and delineates their downstream molecular mechanisms.

**Methods:**

PD models were established using MPTP-treated C57BL/6 mice and MPP^+^-exposed SH-SY5Y neuroblastoma cells. Behavioral assessments (open field, rotarod, and pole tests) and pain sensitivity assays (mechanical allodynia, thermal hyperalgesia) were performed. Molecular analyses included qRT-PCR, Western blot, RNA immunoprecipitation (RIP), co-immunoprecipitation (Co-IP), and ubiquitination assays. Ferroptosis markers (Fe^2+^, ROS, MDA, and GSH) and key regulators (ACSL4, GPX4, SLC7A11, and FTL) were quantified. Gain- and loss-of-function experiments for YTHDF2, CYLD, and NOX4 were conducted to validate regulatory relationships.

**Results:**

MPTP/MPP^+^ treatment downregulated YTHDF2 and upregulated CYLD, exacerbating ferroptosis, as evidenced by mitochondrial damage, elevated Fe^2+^/ROS/MDA, reduced GSH, and altered level of ferroptosis-associated proteins (ACSL4 increased, GPX4/SLC7A11/FTL decreased). YTHDF2 overexpression suppressed ferroptosis, at least in part, by recognizing m6A-modified CYLD mRNA and promoting its degradation. Moreover, CYLD stabilized NOX4 by inhibiting its ubiquitination. Rescue experiments confirmed that CYLD depletion attenuated ferroptosis, an effect that was rescued by NOX4 overexpression. In MPTP-induced mice, YTHDF2 overexpression alleviated motor deficits (improved locomotion, rotarod performance), and reduced pain hypersensitivity, while mitigating ferroptosis markers and nigral mitochondrial pathology.

**Conclusion:**

Our study uncovered the YTHDF2/CYLD/NOX4 axis as a novel ferroptosis regulator in PD, revealing a dual epitranscriptomic-posttranslational therapeutic target for neuroprotection.

**Graphical Abstract:**

Inhibition of YTHDF2-mediated CYLD mRNA decay promotes neuronal ferroptosis and pain in PD via NOX4 deubiquitination.

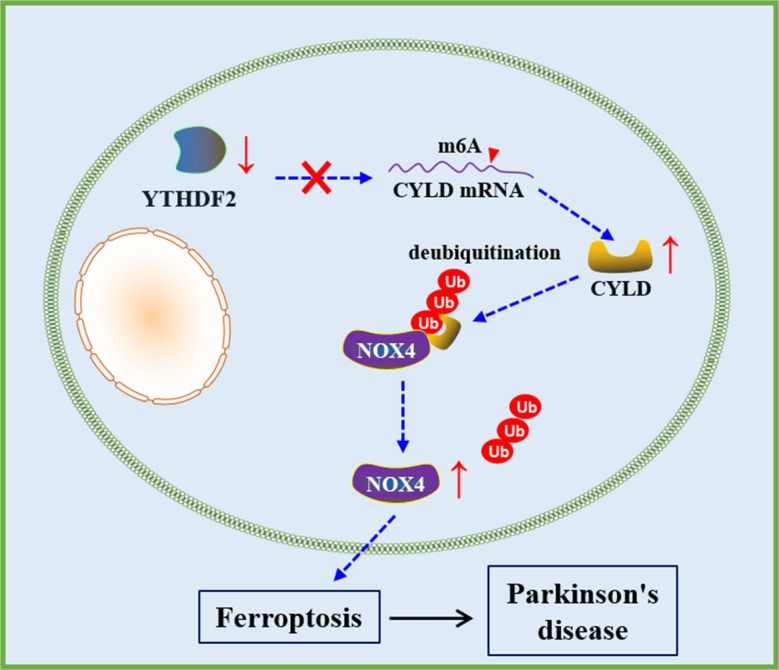

**Supplementary Information:**

The online version contains supplementary material available at 10.1007/s10565-026-10204-0.

## Introduction

Parkinson’s disease (PD) is a progressive neurodegenerative disease caused by dopaminergic neuronal loss in the substantia nigra and is characterized by motor symptoms such as bradykinesia, tremor, rigidity, and postural instability (Fu et al. 2022; Cai et al. 2023). Beyond motor dysfunction, various non-motor manifestations—including cognitive deterioration, mood disturbances, sleep disorders, and pain—substantially contribute to the disease burden (Staunton et al. 2022; Gu et al. 2024). Among these, pain is reported in up to 85% of PD patients and has been widely regarded as a significant factor impairing quality of life (Li et al. 2020). Pain in PD presents as musculoskeletal, radicular-neuropathic, dystonia-induced, akathisia-associated, and centrally-originated forms (Lombardi et al. 2024; Nardelli et al. 2024). However, the management of PD-associated pain remains challenging, as current therapeutic options are often insufficient or only partially effective. Although alterations in dopaminergic pathways have been implicated (Zhang et al. 2023), the pathophysiological mechanisms underlying PD pain are highly complex and not yet fully elucidated. A deeper understanding of the molecular and cellular mechanisms responsible for PD-related pain is urgently needed to discover innovative treatment targets and enhance patient outcomes for affected individuals.

Ferroptosis is a distinct form of regulated cell death marked by iron dependency and the buildup of lipid peroxidation products, fundamentally differing from apoptosis, necrosis, and other cell death pathways (Deng et al. 2025; Cardile et al. 2023). Emerging studies demonstrated that ferroptosis significantly contributes to PD pathogenesis, mainly via iron accumulation and oxidative damage in dopaminergic neurons (Huang et al. 2024). Recent studies have shown that NOX4, a principal source of ROS, exacerbates PD pathology by promoting neuronal ferroptosis and amplifying neuroinflammation (Lin et al. 2025). Furthermore, NOX4 has been linked to neuropathic pain (Geis et al. 2027; Wack et al. 2021; Liu et al. 2023), suggesting its potential involvement in pain processing pathways. However, the function of NOX4 in PD remains insufficiently characterized, and the upstream regulatory mechanisms governing NOX4 activation are still poorly understood. In particular, the specific contribution of NOX4 to PD-associated pain has not yet been elucidated, warranting further investigation to clarify its mechanistic role and therapeutic potential.

CYLD plays a principal function in modulating multiple biological processes, including immune responses, cell proliferation, and apoptosis (Marín-Rubio et al. 2023; Huang and Tan 2023). Previous studies have demonstrated that CYLD level is augmented under conditions of cellular stress, and its activity has been shown to impact the survival of neurons by modulating signaling pathways involved in inflammation and oxidative stress (Romero et al. 2024). For instance, in models of neurodegeneration, CYLD inhibition has been linked to a reduction in oxidative damage and a delay in neuronal cell death, suggesting a protective role against neurodegenerative diseases (Diemert 2012). Specifically, CYLD has been shown to lower PARIS levels, a key factor that induces mitochondrial dysfunction and impairs neuronal survival in PD (Pirooznia et al. 2022; Nardi et al. 2025). By inhibiting CYLD, PARIS expression is reduced, leading to improved mitochondrial function and enhanced bioenergetics, which may contribute to neuronal resilience. However, the specific mechanisms by which CYLD influences PD pathology remain unclear, and further investigation is required. Through database mining and bioinformatic analyses, we identified that NOX4 undergoes ubiquitination modification and may interact with CYLD. Given CYLD’s function as a deubiquitinase, we hypothesize that CYLD may stabilize NOX4 by removing its ubiquitin chains, thereby promoting NOX4 accumulation. Elevated NOX4 levels could, in turn, enhance neuronal ferroptosis and neuroinflammation, contributing to PD development and the progression of PD-associated neuropathic pain. Thus, investigating the CYLD/NOX4 axis may provide new perspectives on PD pathogenesis and identify potential therapeutic targets.

Recent studies have emphasized the importance of N6-methyladenosine (m^6^A) RNA modification in the development and progression of PD (Qiu et al. 2020; He et al. 2023). m^6^A modification, which regulates mRNA stability, splicing, and translation, is mediated by a dynamic balance between "writers" (such as METTL3 and METTL14), "erasers" (such as FTO), and "readers" (such as YTHDF2) (Li et al. 2022; Yan and Fu 2025). In PD patients, the expression levels of METTL3 and METTL14 (He et al. 2023) are significantly reduced, whereas FTO (Li et al. 2025) expression is elevated, suggesting a global disruption of m^6^A homeostasis. Although the contribution of m^6^A dysregulation to PD pathogenesis is increasingly recognized, the specific molecular mechanisms involving YTHDF2 in PD remain largely unknown. Our preliminary analysis identified multiple putative m^6^A modification sites within the CYLD mRNA. Given that YTHDF2 accelerates mRNA degradation by recognizing m^6^A-modified transcripts, we hypothesize that reduced YTHDF2 levels in PD impair the degradation of CYLD mRNA, resulting in its accumulation. These findings suggested a novel epitranscriptomic regulatory pathway contributing to dopaminergic neuron degeneration and pain in PD.

This study aimed to investigate whether YTHDF2 regulates CYLD mRNA stability by recognizing m6A-modified CYLD transcripts, thereby modulating the CYLD/NOX4 axis and influencing ferroptosis-associated changes and pain-related behaviors in PD models.

## Materials and methods

### Experimental animals

Twenty-four male C57BL/6 mice (8–10 weeks old, weighing 20–22 g) were obtained from the Hunan SJA Laboratory Animal Co., Ltd. (Changsha, China). The animals were housed under specific pathogen-free conditions at a controlled temperature of 25 ± 2 °C with 60–70% relative humidity, maintained on a 12-h light/dark cycle. Throughout the experiment, mice received food and water ad libitum. After acclimatization for 1 week, animals were randomly allocated into four groups (n = 6/group): sham control, MPTP model, MPTP + AAV-oe-NC, and MPTP + AAV-oe-YTHDF2.

Mice received daily intraperitoneal administration of MPTP at 25 mg/kg for 7 consecutive days to induce PD-like pathological changes. Mice in the sham group received equal volumes of normal saline. The adeno-associated virus vectors (AAV-oe-YTHDF2 and the corresponding negative control, AAV-oe-NC) were synthesized by Hanheng Biotechnology (Shanghai) Co., Ltd. Two days prior to MPTP administration, mice in the MPTP + AAV-oe-NC and MPTP + AAV-oe-YTHDF2 groups were subjected to stereotactic injection of AAV-oe-NC or AAV-oe-YTHDF2 into both sides of the substantia nigra. For in vivo overexpression, an AAV5 vector driven by the mouse neuron-specific Mecp2 promoter was used. Preliminary validation experiments confirmed that this construct effectively increased YTHDF2 expression in vivo. All mice were subjected to behavioral tests 5 days after the last injection.

### Behavioral tests

All behavioral experiments were carried out and analyzed by researchers who had no access to the grouping information. The rotarod and pole tests are commonly employed for assessing motor function and movement slowness in neurological disease models (Zhi et al. 2025).

Rotarod test: Mice were first trained to stay on the rotarod at a constant speed of 4 revolutions per minute (r/min) until they could maintain balance for more than 2 min. During testing, the rotarod was accelerated from 4 to 40 rpm within 5 min. Fall latency was recorded for each mouse, and the test was repeated three times per animal.

Pole test: A 50-cm-high wooden pole (1 cm in diameter) with a rough surface was vertically positioned in the home cage. During training, mice were placed on top of the pole and allowed to descend to the cage bottom three times. For testing, mice were placed facing downward on the pole top, and the descent time was recorded. Three trials were performed for each animal.

### Open field test

Each mouse was individually placed at the center of an open field arena (40 × 40 × 40 cm) for a 10-min observation period. A video tracking system (Shanghai, China) was used to monitor and analyze locomotor activity and exploratory behavior. After each session, the chamber was cleaned with 75% ethanol to eliminate odor cues. Parameters including movement trajectory, total distance traveled, average speed, and distance covered in the central area were recorded for analysis.

### Immunohistochemistry (IHC)

Mouse brains were transcardially rinsed with ice-cold saline, fixed with 4% paraformaldehyde, and cryoprotected in 30% sucrose before being cut into 25-μm sections on a cryostat. Endogenous peroxidase was blocked with 3% hydrogen peroxide for 10 min, and sections were then washed with PBS and blocked using 1% BSA containing 0.3% Triton X-100. Subsequently, sections were incubated overnight at 4 °C with anti-TH rabbit monoclonal antibody (1:200, #58,844, CST), followed by HRP-conjugated secondary antibody. Signals were developed with a DAB kit (Servicebio), and images were acquired after scanning.

### Immunofluorescence staining

Mouse brains were fixed in 4% paraformaldehyde, dehydrated in 30% sucrose, and sectioned at 25 μm. Sections were blocked with 5% BSA and 0.3% Triton X-100 for 1 h. Primary antibodies against YTHDF2 (1/50, ab246514, Abcam) and NeuN (1/100, ab177487, Abcam) were applied overnight at 4 °C. After washing, sections were incubated with fluorophore-conjugated secondary antibodies for 1 h, and nuclei were stained with DAPI. Images were captured using a fluorescence microscope.

### Mechanical allodynia

Paw withdrawal threshold (PWT) was assessed using Stoelting's von Frey filaments. Mice were acclimated for 30 min in Plexiglass chambers prior to testing. Mechanical stimuli were delivered to the plantar region of the ipsilateral hind paw using von Frey filaments ranging from 0.02 to 4.0 g, and the response threshold was determined with the up-and-down paradigm. A brisk paw withdrawal or licking directed toward the stimulated area was considered a positive nociceptive response. PWT was determined from the response sequence and the force of the final applied filament. Data were log-transformed for statistical analysis.

### Thermal hyperalgesia

Thermal sensitivity was measured using the Hargreaves method. Mice were first acclimated for 30 min in Plexiglass enclosures. An infrared radiometer was then used to determine paw withdrawal latency (PWL) of the ipsilateral hind paw in response to a thermal stimulus. A 30-s cut-off time was applied to prevent thermal injury.

### Electron microscopy

To observe ultrastructural changes, freshly prepared 1 mm-thick SN/STR tissue slices or cell pellets from cultured cells were fixed in 2.5% glutaraldehyde overnight at 4 °C. Samples were then washed three times with 0.1 M PBS and post-fixed in 1% osmium tetroxide for 2 h at 4 °C. After dehydration through a graded ethanol series, samples were embedded in epoxy resin. Randomly selected ultrathin sections were double-stained with uranyl acetate and lead citrate, followed by observation under a TEM (Hitachi).

### Cell culture

HEK293T and SH-SY5Y cell lines were sourced from the Chinese Academy of Sciences Cell Bank and grown in DMEM (VivaCell) containing 10% FBS and 1% penicillin–streptomycin under standard conditions at 37 °C with 5% CO₂. SH-SY5Y cells were cultured under the same conditions and differentiated before subsequent experiments. Briefly, SH-SY5Y cells (passage number ≤ 4) were differentiated by treatment with 1 μM all-trans retinoic acid (RA) for 5 days in complete culture medium containing 1% FBS. After differentiation, the cells were used for subsequent experiments. To establish a PD cellular model, SH-SY5Y were challenged with 1 mM MPP^+^ for 24 h.

The overexpression plasmids for pcDNA3.1-YTHDF2 (oe-YTHDF2), pcDNA3.1-CYLD (oe-CYLD), and pcDNA3.1-NOX4 (oe-NOX4), along with pcDNA3.1 plasmid (oe-NC), as well as short hairpin (sh) RNA targeting YTHDF2 (sh-YTHDF2#1, sh-YTHDF2#2, and sh-YTHDF2#3), CYLD (sh-CYLD), and a negative control (sh-NC), were all purchased from GenePharma (Shanghai, China). Following plasmid or shRNA delivery via Lipofectamine 2000 (Invitrogen), cells were cultured for 48 h.

### CCK-8 assay

SH-SY5Y cells were dispensed into 96-well plates at 2 × 10^3^ cells/well and maintained for 24 h. After another 24 h of incubation, cell viability was assessed by recording the absorbance at 450 nm with a microplate reader.

### Live/dead staining

Treated cells were stained with Calcein-AM/PI (Beyotime) for 30 min and imaged by fluorescence microscopy. Viable cells (green) and dead cells (red) were quantified from three random fields.

### Western blot assay

Total protein was extracted from cells and tissues using RIPA buffer and quantified with a BCA kit (Beyotime, China). Protein samples (30 μg) were electrophoretically separated and transferred to PVDF membranes. Membranes were blocked in 5% non-fat milk and incubated overnight at 4 °C with TH (#58,844, 1:1000, Cell Signaling Technology), YTHDF2 (ab220163, 1:1000, Abcam), ACSL4 (#38,493, 1:1000, Cell Signaling Technology), GPX4 (ab125066, 1:1000, Abcam), FTL (SC0620, 1:5000, Thermo Fisher Scientific), SLC7A11 (PA1-16,893, 1:1000, Thermo Fisher Scientific), CYLD (#43–7700, 1:1000, Thermo Fisher Scientific), NOX4 (MA5-32,090, 1:2000, Thermo Fisher Scientific), and β-actin (MA1-140, 1:5000, Thermo Fisher Scientific)-specific antibodies. Following washing, membranes were incubated with secondary antibodies, and immunoreactive bands were detected using an ECL kit. Band intensities were quantified with ImageJ software.

### qRT-PCR

TRIzol Reagent (Invitrogen) was used to obtain total RNA, which was subsequently converted into cDNA using the High-Capacity cDNA Reverse Transcription Kit (Applied Biosystems). Gene expression was quantified on a LightCycler 480 II system (Roche) with ChamQ Universal SYBR qPCR Master Mix (Vazyme), using GAPDH as the internal control and the 2^−ΔΔCt^ method for analysis. Table 1 presents the primer sequences.

**Table 1:** The primers used in work

Gene	Forward (5’−3’)	Reverse (5’−3’)
YTHDF2(h)	TAGCCAGCTACAAGCACACCAC	CAACCGTTGCTGCAGTCTGTGT
CYLD(h)	TCAGGCTTATGGAGCCAAGAA	ACTTCCCTTCGGTACTTTAAGGA
NOX4(h)	TCTGGCTCT-CCATGAATGTC	CTGCTTGGAACCTTCTGTGA
GAPDH(h)	CTGACTTCAACAGCGACACC	GTGGTCCAGGGGTCTTACTC
YTHDF2(m)	GGTTCTGTGCATCAAAAGGATGG	CCAAAGAATAGGAAAAGCCAATGG
CYLD(m)	GGATGACTCTGCCTGGCTTTTC	CAGGTCCTCCAGAGACATCTTC
GAPDH(m)	AGCCCAAGATGCCCTTCAGT	CCGTGTTCCTACCCCCAATG

###  Intracellular Fe^2+^ content

After washing, cells were incubated with the FerroOrange probe (#F374, Dojindo, Japan) diluted in serum-free medium to a final concentration of 1 μM. After 30 min at 37 °C, fluorescent signals were visualized with an inverted microscope system.

### Measurement of ROS, MDA, GSH

The levels of ROS, MDA, and GSH were tested using the Reactive Oxygen Species Assay Kit (#S0033, Beyotime), MDA Assay Kit (#S0131S, Beyotime), and GSH Assay Kit (#S0053, Beyotime), respectively.

### MeRIP-qPCR

MeRIP-qPCR was conducted using the EpiQuik CUT&RUN m6A RNA Enrichment Kit (Epigentek). Fragmented RNA was enriched with anti-m6A antibody (#202,003, Synaptic Systems) or IgG control through protein A/G magnetic beads, followed by cDNA synthesis and RT-qPCR analysis.

### RIP assay

RIP assays were conducted in HEK293T cells transfected with sh-YTHDF2 or sh-NC. Lysates were incubated overnight at 4 °C with anti-YTHDF2 antibody or IgG control, followed by capture with Protein A/G magnetic beads. The enriched RNAs were purified, reverse-transcribed, and quantified by qPCR for CYLD, with data normalized to Input.

### RNA stability assay

RNA stability was examined by treating sh-NC- or sh-YTHDF2-transfected HEK293T cells with actinomycin D (5 μg/mL; HY-17559, MedChemExpress). CYLD mRNA levels were then quantified by RT-qPCR at the indicated time points.

### Protein stability analysis

Protein degradation was examined in oe-CYLD- or oe-NC-transfected 293 T cells after CHX exposure (10 μg/mL; Sigma-Aldrich) for 0–8 h. Protein abundance was then assessed by Western blot.

### Co-IP analyses

HEK293T cells were used for the ubiquitination-related assays because of their high transfection efficiency, which makes them particularly suitable for plasmid-based protein expression and interaction studies. Cell lysates were prepared in Co-IP buffer and cleared by centrifugation at 13,000 × g for 10 min at 4 °C, followed by overnight incubation with anti-CYLD antibody (#11,110–1-AP, Proteintech) or control IgG (#02–6502, Thermo Fisher), and then with Protein A/G PLUS-Agarose beads (#P2012, Beyotime). After washing, the bound proteins were eluted and analyzed by Western blot for NOX4 and CYLD expression. Additionally, an anti-ubiquitin antibody (#3936, Cell Signaling Technology) was used to evaluate NOX4 ubiquitination levels.

### Statistical analyses

Statistical analyses were performed with SPSS 25.0 and GraphPad Prism 7.0. Differences between two groups were assessed by two-tailed t-tests, and multiple group comparisons by one-way ANOVA with Tukey’s post hoc test. Data are shown as mean ± SEM. A p-value < 0.05 was considered significant.

## Results

### YTHDF2 was decreased and CYLD was enhanced in MPTP-induced PD mice

A murine model of MPTP-stimulated PD was established to probe the role of YTHDF2 and CYLD in PD. Mouse body weight was recorded daily at a fixed time during the experiment. The MPTP group had lower body weight relative to the sham group, though no statistically significant difference was observed (Figure S1**A**). MPTP-stimulated mice exhibited a distinct suppression in total movement distance, decreased average speed, and prolonged immobility time in the open field test (Figure S1B). Besides, MPTP-triggered mice exhibited impaired pole-climbing ability and significantly reduced rotarod retention time (Figure S1C and D). We measured the TH expression in striatum and brain tissues and found that it was decreased in PD mice compared with sham mice (Figure S1E and F). Further, MPTP-induced PD mice exhibited distinct depletion of YTHDF2 and upregulation of CYLD in the striatum (Figure S1G). These findings suggest that downregulation of YTHDF2 and upregulation of CYLD may underlie the development of MPTP-induced PD.

### YTHDF2 was downregulated and CYLD was upregulated in MPP^+^-triggered SH-SY5Y cells

**Fig. 1:**
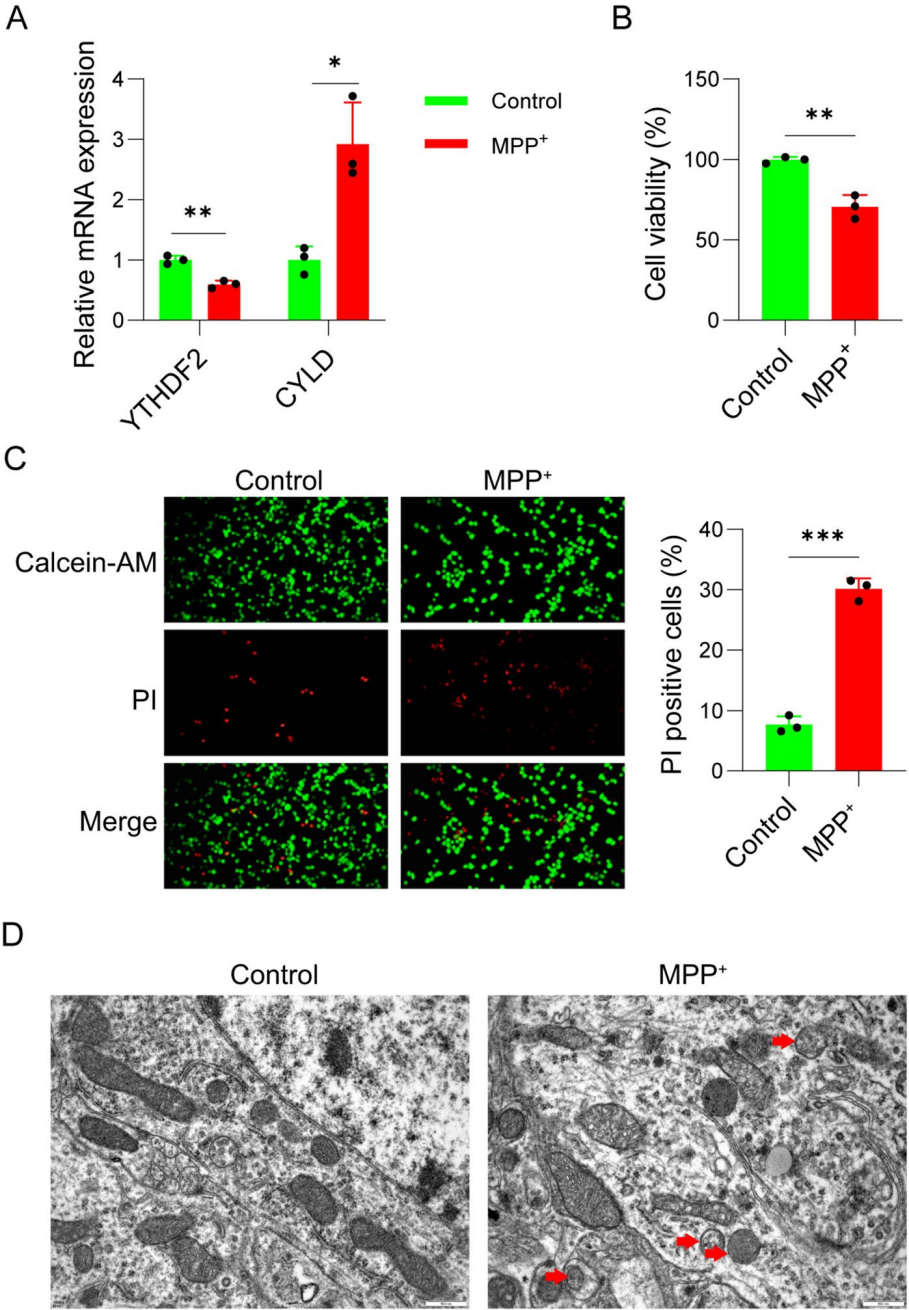
YTHDF2 was downregulated and CYLD was upregulated in MPP^+^-triggered SH-SY5Y cells. SH-SY5Y cells were divided into two groups: Control and MPP^+^. **A** The mRNA expression levels of YTHDF2 and CYLD were analyzed by qRT-PCR. **B** Cell viability was assessed using the CCK-8 assay. **C** Neuronal viability was evaluated by live/dead staining with Calcein-AM (green, viable cells) and propidium iodide (PI, red, dead cells). **D** Transmission electron microscopy (TEM) revealed ferroptosis-like mitochondrial morphological changes, which were interpreted in conjunction with biochemical ferroptosis-related markers. n = 3. *p < 0.05, **p < 0.01, ***p < 0.001

To mimic PD in vitro, SH-SY5Y cells were challenged with 1 mM MPP^+^ for 24 h. As unveiled in Fig. 1A, YTHDF2 expression was downregulated, while CYLD level was upregulated in MPP^+^-exposed cells. In addition, cell viability was significantly reduced following MPP^+^ exposure (Fig. 1B). To further evaluate cell damage, Calcein-AM/PI co-staining was utilized. As expounded in Fig. 1C, MPP^+^ treatment increased the number of dead cells and diminished the number of live cells. Moreover, ferroptosis was observed in MPP^+^-exposed SH-SY5Y cells, as evidenced by mitochondrial fragmentation, swelling, vacuolization, and loss of mitochondrial structural integrity (Fig. 1D). These findings collectively confirm the successful establishment of a PD-like cellular model and suggest that MPP^+^ induces ferroptosis in SH-SY5Y cells.

###  Overexpression of YTHDF2 suppressed ferroptosis in MPP^+^ -triggered cells

**Fig. 2:**
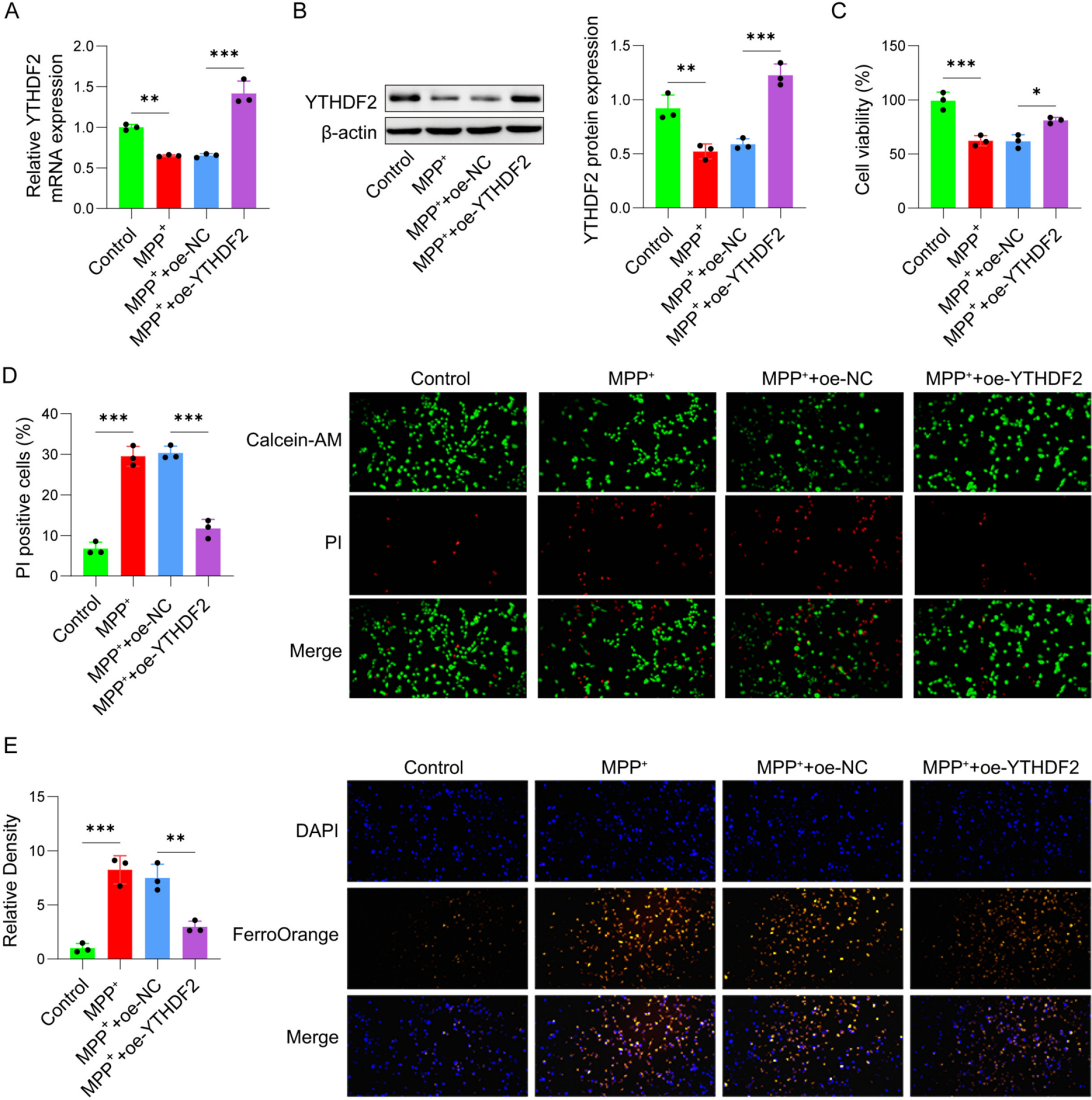

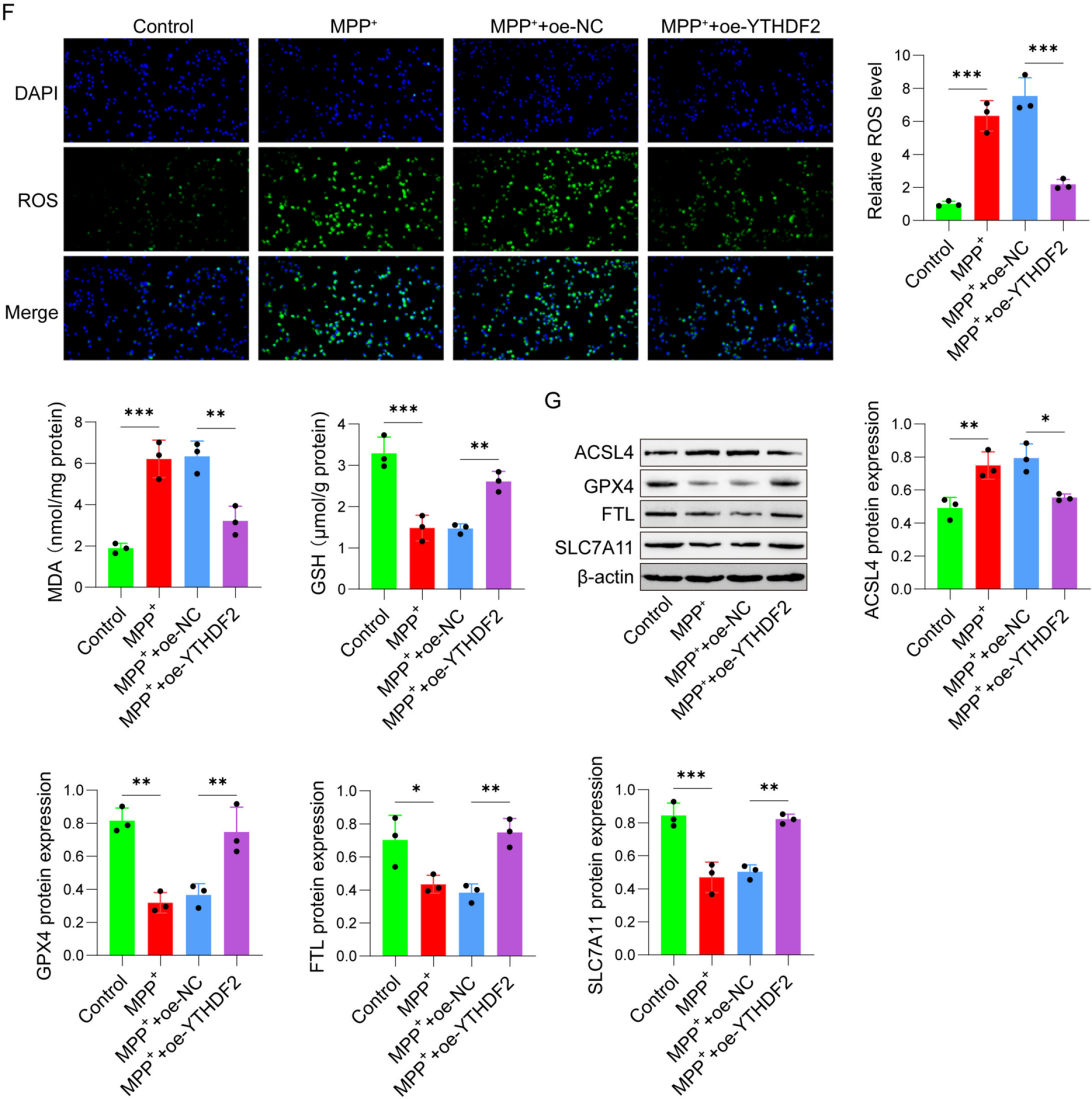
Overexpression of YTHDF2 suppressed ferroptosis in MPP^+^-triggered SH-SY5Y cells. SH-SY5Y cells were divided into four groups: Control, MPP^+^, MPP^+^ + oe-NC, and MPP^+^ + oe-YTHDF2. **A** and **B** YTHDF2 mRNA and protein levels were measured by qRT-PCR and western blot assay. **C** CCK-8 assay was utilized to measure cell viability. **D** Cell viability was evaluated by live/dead staining (Calcein-AM [green, viable cells] and propidium iodide [PI, red, dead cells]). **E** Intracellular Fe^2^⁺ accumulation was detected using FerroOrange fluorescence probe. **F** The levels of ROS, MDA, and GSH were detected according to the instructions of the reagent kit, respectively. **G** ACSL4, GPX4, FTL, and SLC7A11 protein levels were detected by western blot assay. n = 3. *p < 0.05, **p < 0.01, ***p < 0.001

YTHDF2 levels were decreased in MPP^+^-triggered SH-SY5Y cells, an effect that was reversed by YTHDF2 overexpression (Fig. 2A and B). As depicted in Fig. 2C, YTHDF2 addition reversed the MPP^+^-caused diminution of cell viability. Moreover, MPP^+^ treatment increased the number of dead cells and diminished the number of live cells, effects that were reversed by YTHDF2 overexpression (Fig. 2D). Moreover, MPP^+^ treatment significantly elevated Fe^2+^ (Fig. 2E), ROS (2F), and MDA (2F) levels, while reducing GSH (Fig. 2F) levels in SH-SY5Y cells; these effects were significantly attenuated by YTHDF2 overexpression. Besides, MPP^+^ treatment upregulated ACSL4 expression while downregulating GPX4, FTL, and SLC7A11 levels in SH-SY5Y cells. These alterations were partially reversed by YTHDF2 overexpression (Fig. 2G). These findings indicate that overexpression of YTHDF2 inhibits MPP^+^-induced ferroptosis in vitro. Cell viability was comparable among the Control, oe-NC, and sh-NC groups (Figure S2A), intracellular Fe^2^⁺ accumulation (Figure S2B), ROS levels, MDA content, or GSH levels (Figure S2C) under basal conditions, indicating that the empty vector and negative control shRNA did not affect cellular phenotypes.

### YTHDF2 promoted the degradation of CYLD mRNA

**Fig. 3:**
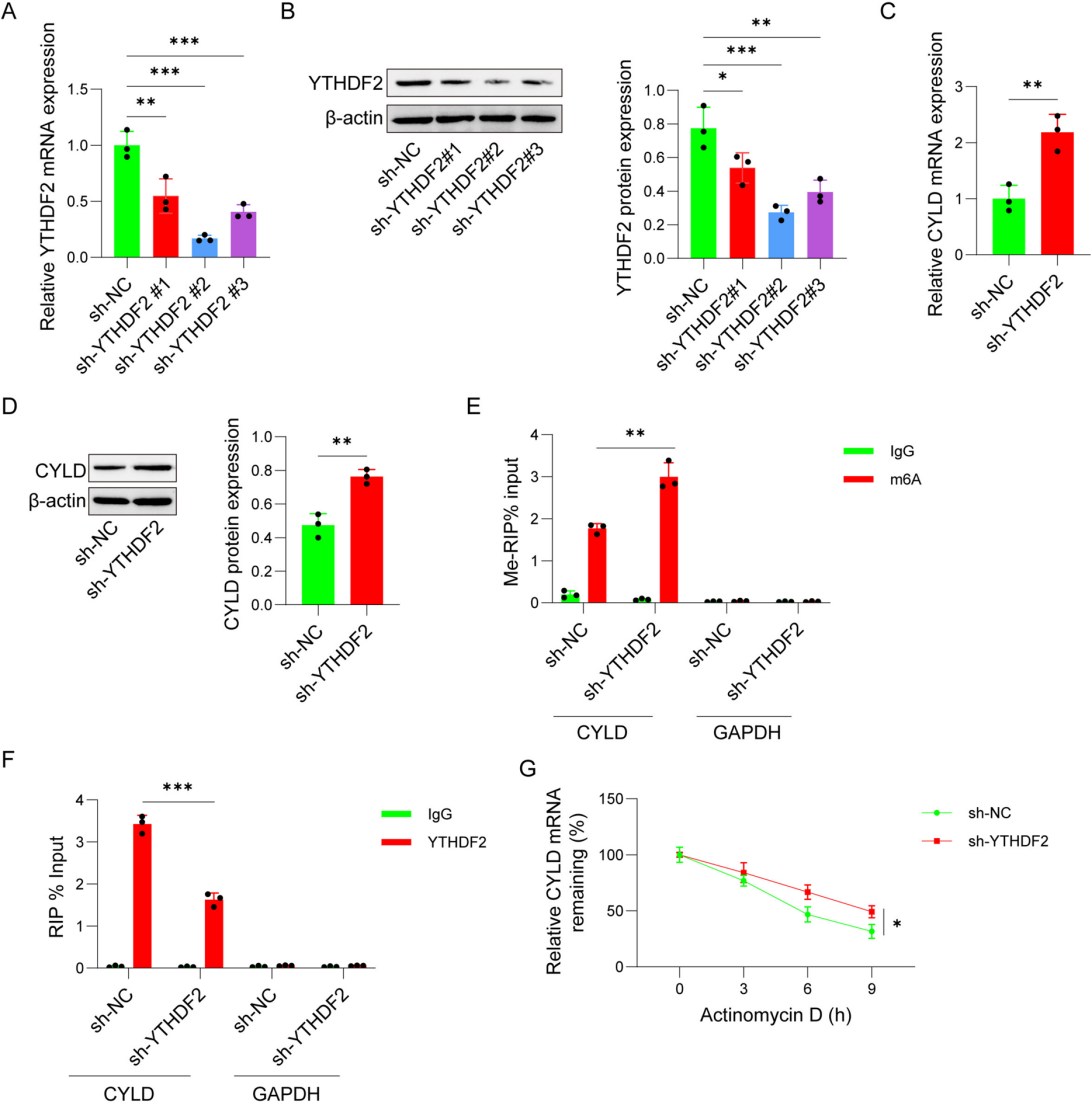
YTHDF2 promoted the degradation of CYLD mRNA. **A** and **B** Three YTHDF2 shRNAs (sh-YTHDF2#1, #2, and #3) effectively reduced YTHDF2 expression, as confirmed by qRT-PCR and western blot assays. **C** and **D** CYLD mRNA and protein levels were detected by qRT-PCR and western blot assays. **E** MeRIP-qPCR analysis of CYLD mRNA enrichment, with enrichment normalized to input. **F** RIP was used to analyze the enrichment level of YTHDF2 on CYLD mRNA. **G** For RNA stability analysis, sh-NC and sh-YTHDF2 293 T cells were incubated with actinomycin D. n = 3. *p < 0.05, **p < 0.01, ***p < 0.001

Three YTHDF2-targeting shRNAs (shRNA#1, #2, and #3) effectively knocked down YTHDF2 expression, with sh-YTHDF2#2 showing the strongest silencing effect and thus being used in later experiments (Fig. 3A and B). As an m^6^A-binding protein, YTHDF2 is known to accelerate mRNA decay by recognizing m^6^A-modified transcripts and promoting transcript degradation (Tan et al. 2022; Du et al. 2020). Consistent with its role in mRNA destabilization, knockdown of YTHDF2 increased CYLD levels (Fig. 3C and D). Moreover, MeRIP-qPCR showed increased enrichment of m^6^A-modified CYLD mRNA in the YTHDF2-silenced group (Fig. 3E). To further verify the direct effect of YTHDF2 on CYLD mRNA, RIP assay demonstrated that YTHDF2 directly interacts with CYLD mRNA, and its knockdown markedly decreased the enrichment level of CYLD mRNA in the immunoprecipitates (Fig. 3F). Depletion of YTHDF2 significantly attenuated CYLD mRNA degradation, thereby increasing its transcript stability (Fig. 3G). These data indicated that YTHDF2 regulates CYLD expression by recognizing m6A-modified CYLD mRNA and promoting its degradation, thereby contributing to post-transcriptional control of CYLD expression.

To further validate whether CYLD mRNA undergoes m6A modification under PD-like conditions, we performed MeRIP-qPCR analysis in SH-SY5Y cells following MPP^+^ exposure. As depicted in Figure S3, MPP^+^ treatment visibly increased the m6A enrichment level of CYLD mRNA compared with the control group, further supporting that CYLD is an m6A-modified transcript in the MPP^+^-treated PD cellular model.

###  YTHDF2 suppressed ferroptosis via regulating CYLD in MPP^+^-exposed cells

**Fig. 4:**
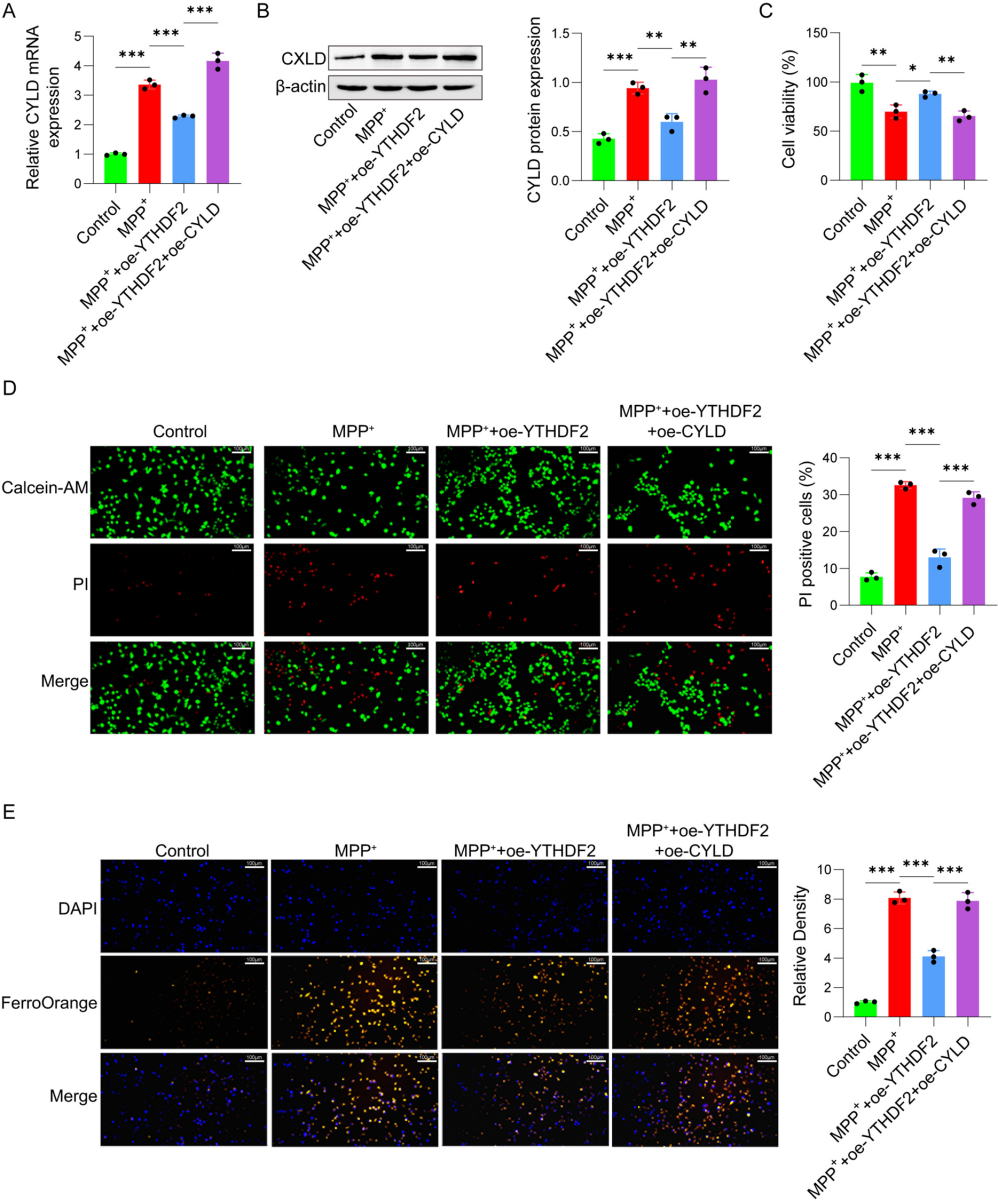

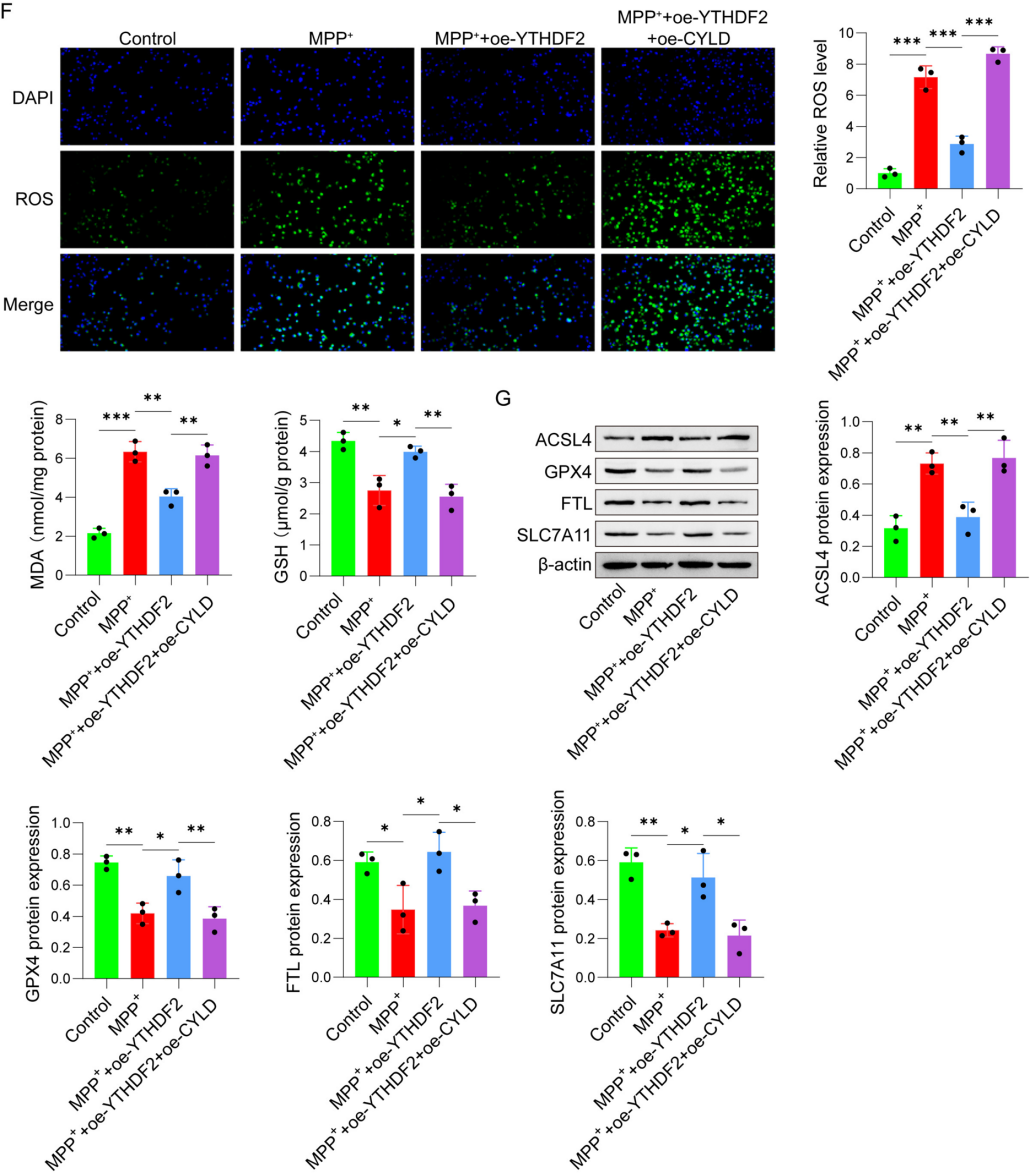
YTHDF2 suppressed ferroptosis via regulating CYLD in MPP^+^-triggered SH-SY5Y cells. Experiments were divided into four groups: Control, MPP^+^, MPP^+^ + oe-YTHDF2, and MPP^+^ + oe-YTHDF2 + oe-CYLD. **A** and **B** CYLD mRNA and protein levels were detected by qRT-PCR and western blot assays. **C** CCK-8 assay was utilized to measure cell viability. **D** Neuronal viability was measured by using Calcein-AM and PI dyes. **E** Detection of intracellular Fe^2+^ with FerroOrange. **F** The levels of ROS, MDA, and GSH were detected according to the instructions of the reagent kit, respectively. **G** ACSL4, GPX4, FTL, and SLC7A11 protein levels were detected by western blot assay. n = 3. *p < 0.05, **p < 0.01, ***p < 0.001

To investigate whether YTHDF2 mediates ferroptosis in cells through CYLD, SH-SY5Y cells were assigned to the following groups: Control, MPP^+^, MPP^+^ + oe-YTHDF2, and MPP^+^ + oe-YTHDF2 + oe-CYLD. In MPP^+^-exposed SH-SY5Y cells, YTHDF2 downregulated CYLD expression, an effect that was reversed upon CYLD overexpression (Fig. 4A and B). MPP^+^ treatment decreased cell viability, which was partially rescued by YTHDF2 overexpression; however, this rescue effect was reversed by co-overexpression of CYLD (Fig. 4C). Besides, MPP^+^ led to an augmentation in dead cells and a decline in viable cells. Addition of YTHDF2 partially restored cell proliferation, while co-overexpression of CYLD reversed this effect (Fig. 4D). Moreover, YTHDF2 overexpression decreased the levels of Fe^2+^ (Fig. 4E), ROS (4F), and MDA (4F), and elevated GSH (Fig. 4F) levels; however, these changes were reversed upon CYLD overexpression. Further, MPP^+^ treatment increased ACSL4 expression but decreased GPX4, FTL, and SLC7A11 levels in SH-SY5Y cells. Addition of YTHDF2 partially reversed these effects, whereas CYLD overexpression abolished the rescue mediated by YTHDF2 (Fig. 4G). These data displayed that YTHDF2 modulates ferroptosis in MPP^+^ -treated SH-SY5Y cells, at least in part, by recognizing m6A-modified CYLD mRNA and promoting its degradation.

### CYLD stabilized NOX4 protein by inhibiting NOX4 ubiquitination

**Fig. 5:**
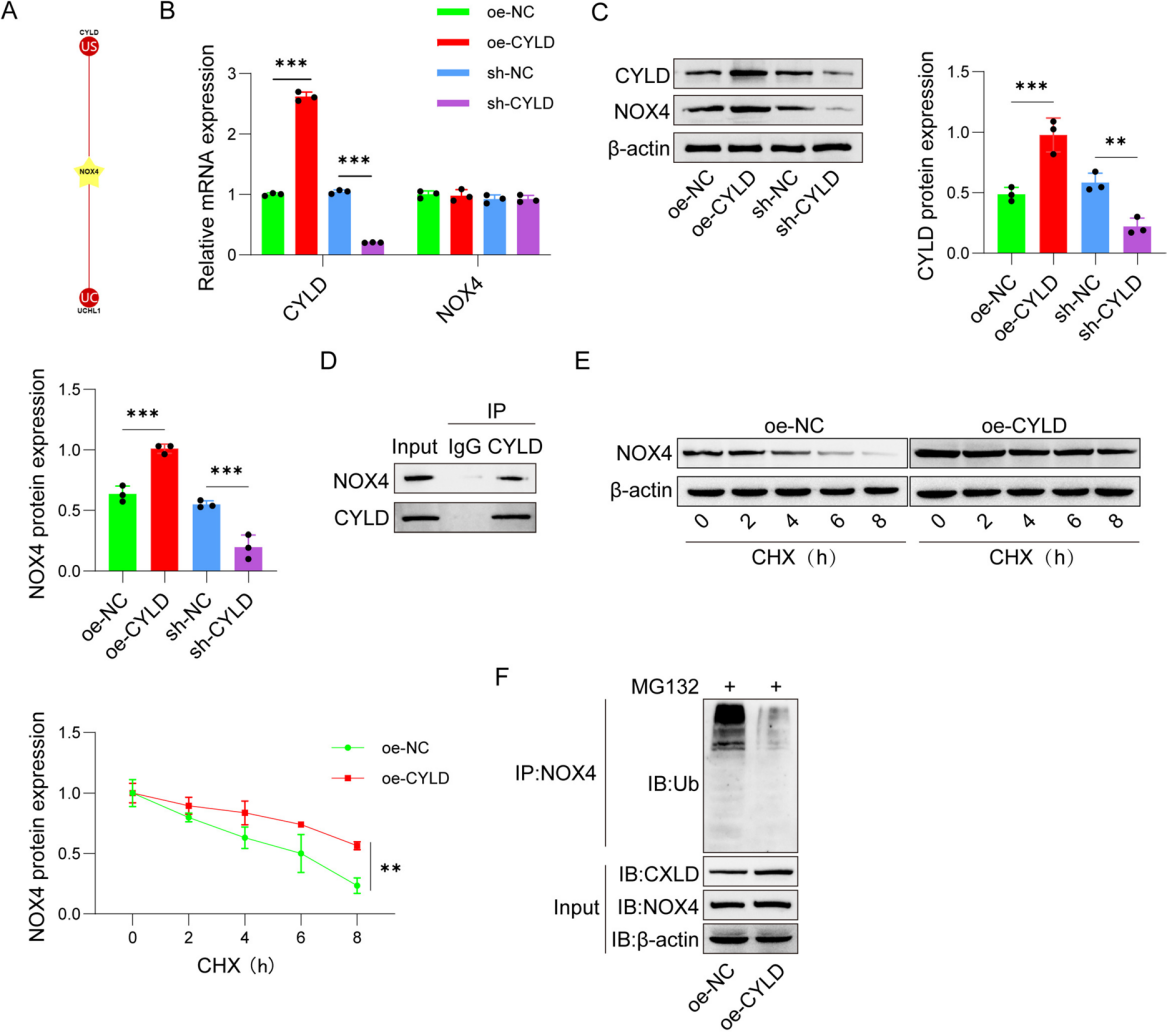
CYLD stabilized NOX4 protein by inhibiting NOX4 ubiquitination. **A** Bioinformatic analysis was performed using the UbiBrowser platform to identify potential deubiquitinases targeting NOX4. **B** and **C** CYLD and NOX4 mRNA and protein levels were measured by qRT-PCR and western blot assay. **D** The endogenous interaction between CYLD and NOX4 was measured by Co-IP assay. **E** Representative blots and quantification showed knockout of CYLD enhanced the stability of NOX4. **F** NOX4 ubiquitination was measured in CYLD-depleted 293 T cells. n = 3. **p < 0.01, ***p < 0.001

Subsequently, we investigated the underlying mechanism by which the deubiquitinase CYLD regulates neuronal ferroptosis. The UbiBrowser analysis identified the deubiquitinase CYLD as a potential regulator of NOX4 (Fig. 5A). CYLD was overexpressed or silenced in 293 T cells to evaluate its effect on NOX4. Increased CYLD elevated NOX4 protein, but not NOX4 mRNA, while CYLD silencing showed the opposite trend (Fig. 5B and C). Besides, Co-IP assay results indicated that the endogenous NOX4 protein was coprecipitated by a CYLD-specific antibody (Fig. 5D). Further, CYLD overexpression stabilized NOX4 protein levels in 293 T cells (Fig. 5E). Notably, CYLD overexpression decreased NOX4 ubiquitination in 293 T cells (Fig. 5F). Notably, to determine which ubiquitin linkage type was affected by CYLD, we further assessed K48- and K63-linked ubiquitination of NOX4. The results indicated that CYLD obviously decreased K48-linked ubiquitination of NOX4, while exerting no obvious effect on K63-linked ubiquitination (Figure S4). These data further implied that CYLD stabilizes NOX4 mainly through K48-linked deubiquitination.

###  CYLD mediated ferroptosis through NOX4 in MPP ^+^ -treated cells

**Fig. 6:**
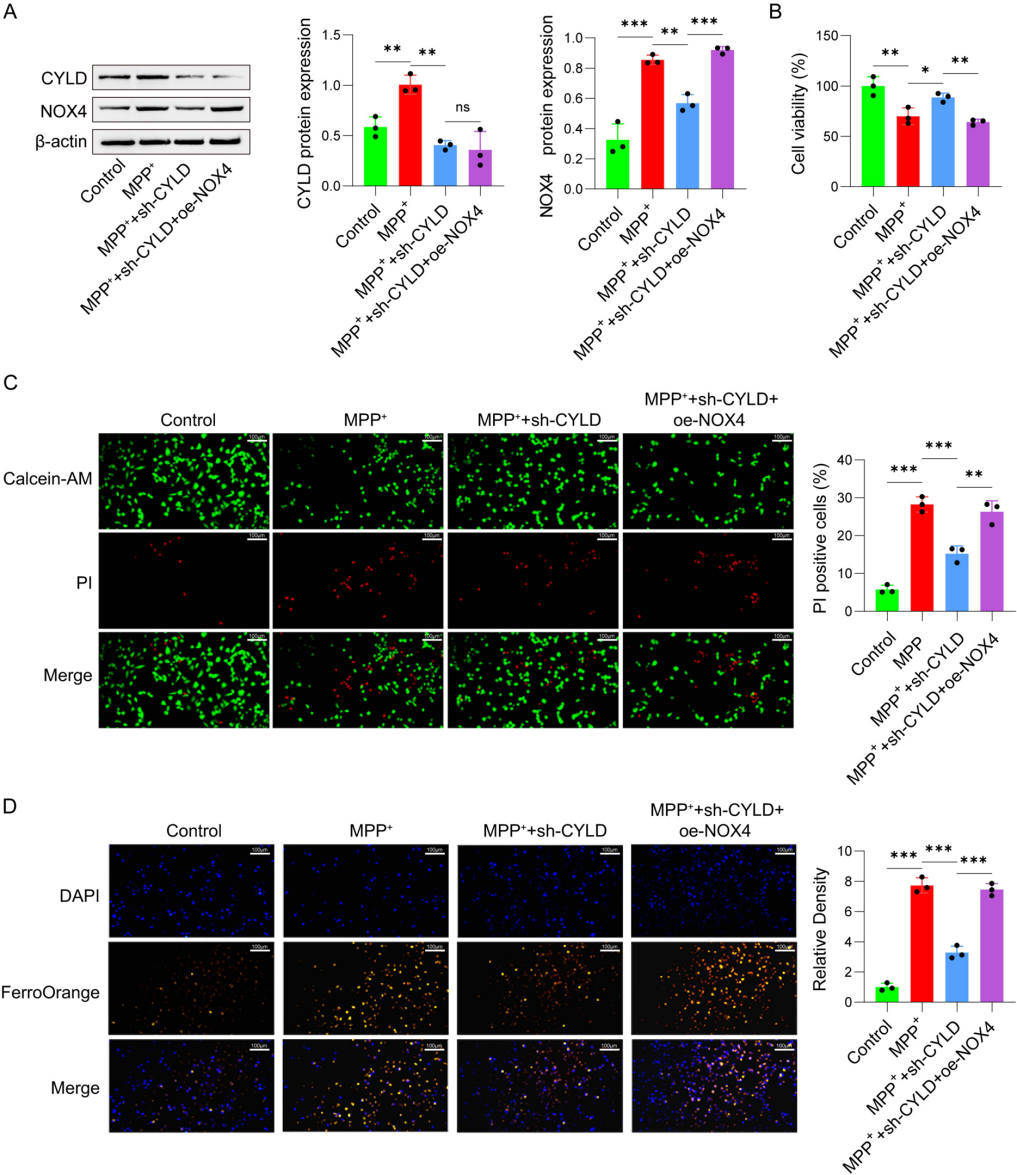

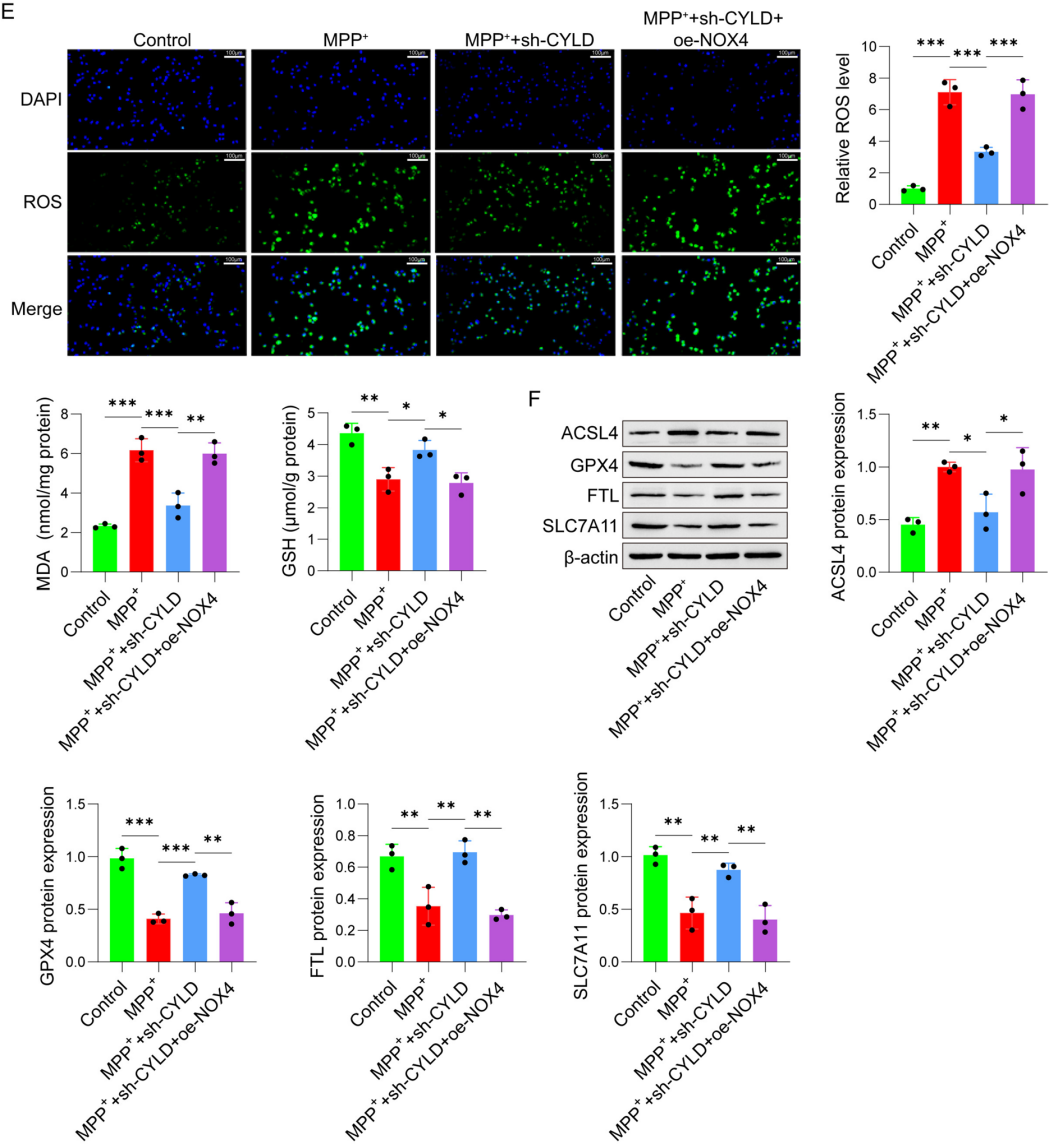
CYLD mediated ferroptosis through NOX4 in MPP^+^-triggered SH-SY5Y cells. SH-SY5Y cells were divided into four groups: Control, MPP^+^, MPP^+^ + sh-CYLD, and MPP^+^ + sh-CYLD + oe-NOX4. **A** CYLD and NOX4 protein levels were measured by western blot assay. **B** CCK-8 assay was utilized to measure cell viability. **C** Neuronal viability was measured by using Calcein-AM and PI dyes. **D** Detection of intracellular Fe^2+^ with FerroOrange. **E** The levels of ROS, MDA, and GSH were detected according to the instructions of the reagent kit, respectively. **F** ACSL4, GPX4, FTL, and SLC7A11 protein levels were detected by western blot assay. n = 3. *p < 0.05, **p < 0.01, ***p < 0.001

To explore whether CYLD mediates ferroptosis in SH-SY5Y cells through NOX4, cells were assigned to the following groups: Control, MPP^+^, MPP^+^ + sh-CYLD, and MPP^+^ + sh-CYLD + oe-NOX4. In MPP^+^-stimulated SH-SY5Y cells, CYLD knockdown reduced both CYLD and NOX4 expression levels, whereas NOX4 overexpression specifically restored NOX4 expression (but not CYLD expression) (Fig. 6A). Besides, MPP^+^ treatment decreased cell viability, which was partially rescued by CYLD silencing; however, this rescue effect was abolished by NOX4 overexpression (Fig. 6B). Additionally, MPP^+^ treatment increased the number of dead cells and reduced viable cells, with CYLD silencing partially restoring cell proliferation. Co-overexpression of NOX4 reversed this effect (Fig. 6C). Furthermore, CYLD silencing in MPP^+^-stimulated cells decreased the levels of Fe^2+^ (Fig. 6D), ROS (6E), and MDA (6E), while increasing GSH levels (Fig. 6E); these changes were reversed upon NOX4 overexpression. MPP^+^ treatment also upregulated ACSL4 level and downregulated GPX4, FTL, and SLC7A11 levels in SH-SY5Y cells. CYLD silencing partially reversed these effects, whereas NOX4 overexpression abolished the rescue effect induced by CYLD silencing (Fig. 6F). These results recommended that CYLD silencing suppressed ferroptosis in MPP^+^-treated SH-SY5Y cells through regulation of NOX4.

### YTHDF2 alleviated neuronal ferroptosis-associated alterations and pain-related behaviors in PD mouse models, accompanied by regulation of the CYLD/NOX4 axis

**Fig. 7:**
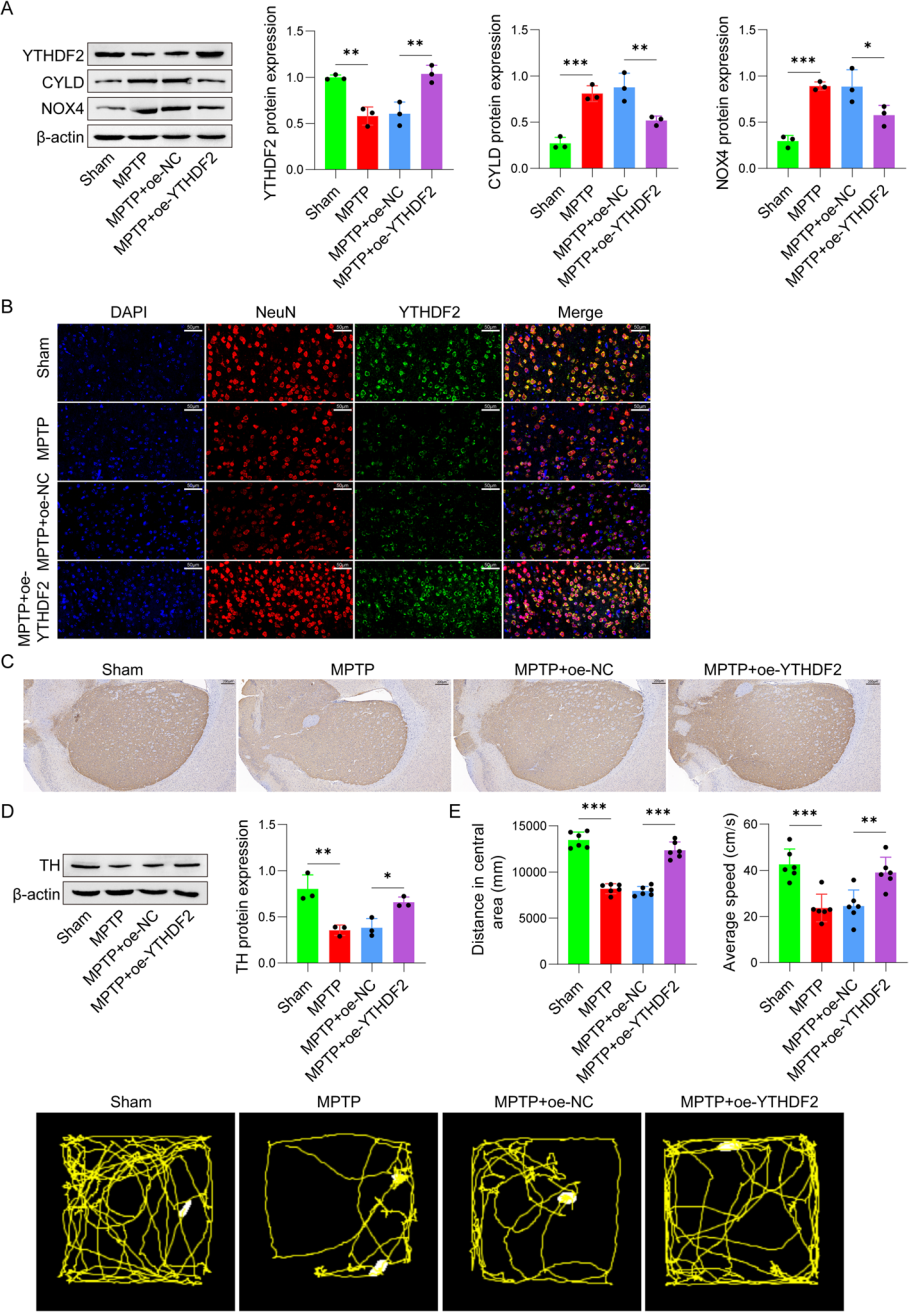

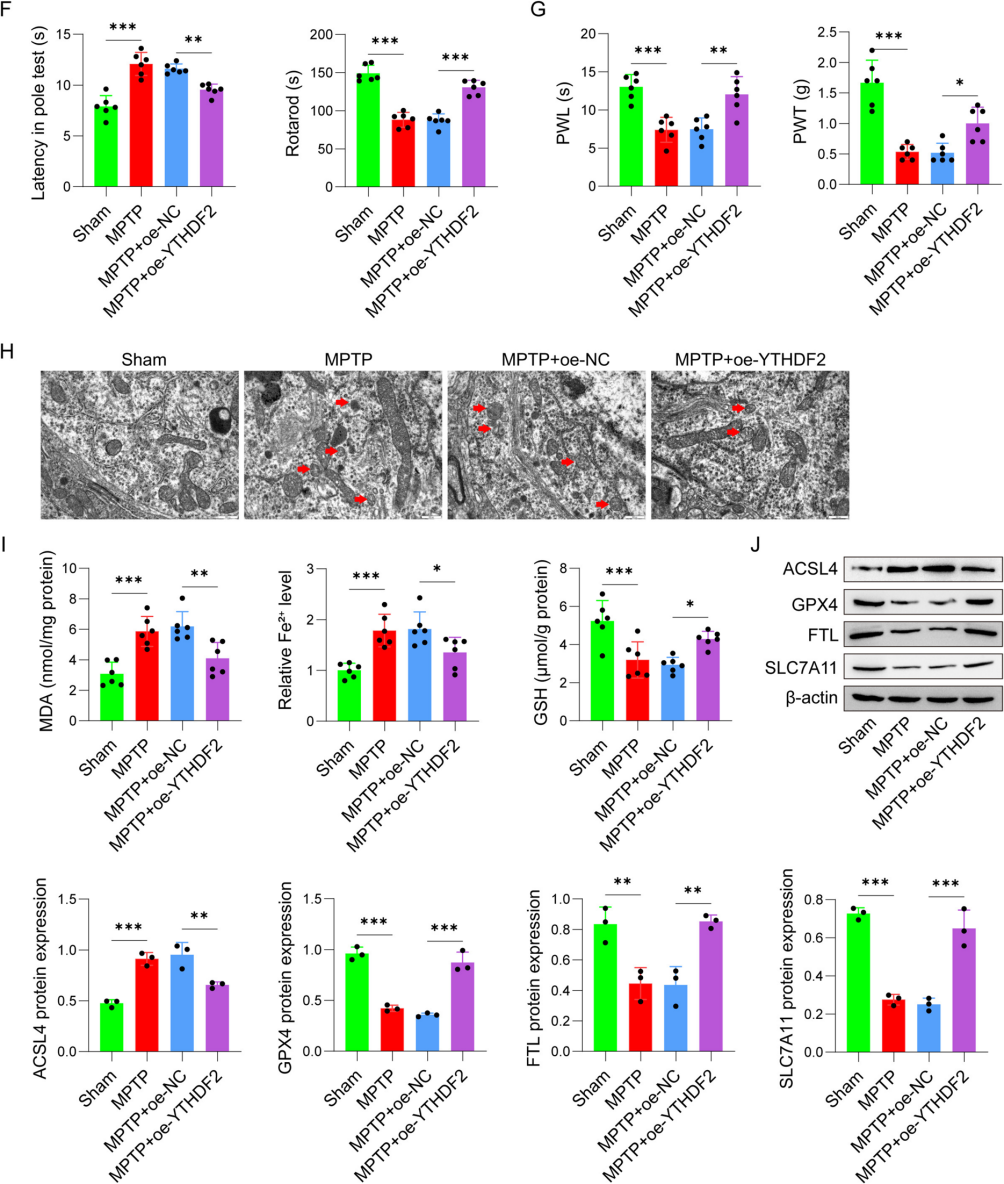
YTHDF2 alleviated neuronal ferroptosis-associated alterations and pain-related behaviors in PD mouse models, accompanied by regulation of the CYLD/NOX4 axis. Mice were divided into four groups: Sham, MPTP, MPTP + AAV-oe-NC, and MPTP + AAV-oe-YTHDF2. **A** Western blot analysis of YTHDF2, CYLD, and NOX4 protein levels in mouse brain tissue. **B** Immunofluorescence staining of YTHDF2 and NeuN in the striatum. **C** Immunohistochemical staining of tyrosine hydroxylase (TH) in the striatum. **D** Western blot detection of TH protein in brain tissue. **E** Open field test assessing locomotor activity, including center zone crossings and average speed. **F** Behavioral assessment using the pole test and rotarod test to evaluate motor coordination and balance. **G** Mechanical allodynia and thermal hyperalgesia tests to evaluate mechanical and thermal sensitivity. **H** TEM images of the substantia nigra showing ferroptosis-like mitochondrial morphological changes, which were interpreted in conjunction with biochemical ferroptosis-related markers. **I** Biochemical assays measuring MDA, GSH, and Fe^2+^ levels in brain tissue. **J** Western blot analysis of ferroptosis-related proteins including ACSL4, GPX4, FTL, and SLC7A11. n = 6. *p < 0.05, **p < 0.01, ***p < 0.001

Mice were subjected to stereotactic injection of AAV-oe-NC or AAV-oe-YTHDF2, AAV-oe-YTHDF2 effectively increased YTHDF2 expression in vivo (Figure S5). In MPTP-induced mice, YTHDF2 expression was significantly downregulated, accompanied by upregulation of CYLD and NOX4. Overexpression of YTHDF2 reversed these changes (Fig. 7A). Meanwhile, YTHDF2 levels in the striatum, reduced by MPTP, were restored following YTHDF2 overexpression (Fig. 7B). MPTP treatment also led to a marked decline in TH expression, both in the striatum and brain tissue, which was alleviated by YTHDF2 overexpression (Fig. 7C and D). Behaviorally, MPTP-treated mice exhibited reduced center crossings and average velocity in the open field test, prolonged pole descent time, and shortened rotarod retention time, all of which were improved by YTHDF2 overexpression (Fig. 7E and F). Pain sensitivity was also altered, as evidenced by decreased PWT and PWL in the MPTP group, effects that were reversed with YTHDF2 overexpression (Fig. 7G). Morphologically, mitochondrial shrinkage, cristae loss, and outer membrane rupture in the substantia nigra indicated ferroptosis, which was mitigated by YTHDF2 (Fig. 7H). Biochemically, MPTP increased MDA and Fe^2+^ levels while decreasing GSH levels (Fig. 7I), and also upregulated ACSL4 while downregulating GPX4, FTL, and SLC7A11 (Fig. 7J); these ferroptosis-related alterations were partially reversed by YTHDF2 overexpression. Collectively, these findings implied that YTHDF2 alleviates MPTP-induced neurodegeneration and ferroptosis-associated alterations, while improving pain-related behavioral outcomes.

## Discussion

PD is marked by progressive degeneration of dopaminergic neurons and a range of non-motor symptoms, including chronic pain, which severely affect patients' quality of life (Rei et al. 2024; Tedeschi 2023; Yang et al. 2023). Although accumulating evidence has highlighted the importance of m^6^A RNA modification in PD pathogenesis (Miller et al. 2025; Liang et al. 2025), the specific molecular mechanisms linking m^6^A dysregulation to neuronal ferroptosis and neuropathic pain have remained elusive. In this work, we demonstrated that the decrease in YTHDF2 expression in PD impairs CYLD mRNA degradation, leading to elevated CYLD expression. Our findings revealed a novel m6A/YTHDF2/CYLD/NOX4 signaling axis associated with neurodegeneration, ferroptosis-related alterations, and pain-related behaviors in PD models, providing novel perspectives on the molecular mechanisms underlying disease progression and identifying potential therapeutic targets for intervention.

Increasing evidence links ferroptosis—a lipid peroxidation-driven, iron-reliant cell death pathway—to PD development (Zhao et al. 2023; Wang et al. 2022). Emerging evidence suggested that iron accumulation, GPX4 dysfunction, and excessive lipid peroxidation contribute to dopaminergic neuronal loss in PD models (Chu et al. 2023). Studies have shown that ferroptosis inhibitors, such as ferrostatin-1, can alleviate neurodegeneration in PD, further supporting the importance of ferroptosis in PD progression (Tang et al. 2023). Consistent with these findings, our study provided compelling evidence that ferroptosis was robustly activated in PD models. This was supported by a marked increase in key ferroptotic markers, including iron accumulation, ROS, and MDA levels, as well as upregulated ACSL4 expression. Conversely, we observed significant downregulation of ferroptosis defense mechanisms, manifested by reduced GSH levels and reduced expression of GPX4, FTL, and SLC7A11. These findings support a central role of ferroptosis in PD-related neuronal injury and identify ferroptosis modulation as a possible therapeutic strategy.

Growing evidence suggests that m6A RNA methylation is closely involved in neuronal function and neurodegenerative processes (Shu et al. 2021). Dysregulation of m6A modification and its key regulators, such as METTL3, METTL14, and FTO, has been implicated in PD pathogenesis (Deng et al. 2022; Wang et al. 2020; Yang et al. 2019). Among m^6^A readers, YTHDF2 is known to selectively bind m^6^A-modified transcripts and promote their degradation, thereby fine-tuning gene expression post-transcriptionally (Chen et al. 2024). In the nervous system, increasing evidence suggested that YTHDF2 exerts protective effects against neurodegeneration and injury (Huang et al. 2024b). Existing research has shown that YTHDF2-mediated mRNA degradation is required for neuronal differentiation, axonal regeneration, and synaptic plasticity (Zhuang et al. 2023). For instance, YTHDF2 has been reported to facilitate axon regeneration after nerve injury by selectively degrading negative regulators of axon growth (Li et al. 2018). Furthermore, in models of ischemic stroke, YTHDF2 alleviates neuronal apoptosis and reduces infarct volume through modulating m^6^A-dependent transcript stability (Chen et al. 2024b). Recent research also indicates that YTHDF2 can mitigate inflammatory responses in microglia, thus providing neuroprotection in neuroinflammatory conditions (Pan et al. 2021). Collectively, these findings emphasized the beneficial role of YTHDF2 in preserving neuronal integrity and function, suggesting that impairment of YTHDF2-mediated mRNA regulation may contribute to the pathogenesis of various neurological disorders. In the present work, we demonstrated that YTHDF2 level was markedly decreased in PD models, suggesting a potential link between impaired m^6^A-dependent mRNA decay and neuronal injury in PD. Addition of YTHDF2 inhibited ferroptosis in MPP^+^-triggered SH-SY5Y cells. It is worth noting that YTHDF2 also alleviated ferroptosis-associated alterations and improved pain-related behaviors in MPTP-induced PD mice. Importantly, our data support a model in which YTHDF2 recognizes m^6^A-modified CYLD mRNA and facilitates its degradation, thereby suppressing neuronal ferroptosis. Importantly, although YTHDF2 is known to exert broad post-transcriptional regulatory effects, our data support that the CYLD/NOX4 axis represents a major functional pathway underlying the regulatory role of YTHDF2 in PD-related ferroptosis. Given the broad regulatory nature of m6A modification and YTHDF2-mediated RNA decay, we cannot exclude the possibility that additional ferroptosis-related transcripts may also be involved under PD-like conditions. However, our findings identified CYLD as a necessary downstream effector of YTHDF2 and further demonstrated that CYLD promotes ferroptosis, at least in part, by stabilizing NOX4 protein through deubiquitination. Thus, CYLD served as an important mechanistic hub linking epitranscriptomic regulation to NOX4-dependent oxidative stress in PD.

The involvement of deubiquitinating enzymes in PD pathogenesis has received growing attention, particularly in the context of neuronal vulnerability (Lee et al. 2020). CYLD, initially recognized as an anticancer regulator, is increasingly associated with neurodegenerative disease progression (Nardi et al. 2025; Sun 2010). In the central nervous system, CYLD modulates key pathways, including autophagy, NF-κB, and Wnt/β-catenin, which are critical for neuronal survival and homeostasis (Nardi et al. 2025; Mathis et al. 2015). CYLD dysregulation may aggravate neuroinflammatory responses, disrupt mitochondrial homeostasis, and amplify oxidative stress, thereby contributing to neurodegenerative pathology (Schramm et al. 2024). For example, studies have shown that CYLD exacerbates dopaminergic neuron loss in models of PD by promoting mitochondrial dysfunction and inhibiting mitophagy (Pirooznia et al. 2022). Moreover, CYLD has been reported to contribute to axonal degeneration and neuroinflammatory responses following traumatic brain injury (Nardi et al. 2025). These findings suggested that CYLD acts as a detrimental regulator in the nervous system, and its abnormal activation may amplify neuronal vulnerability under pathological conditions. Here, we found that CYLD level was enhanced in animal and cellular PD models. Consistent with the results of previous studies, CYLD aggravated ferroptosis and promoted the progression of PD.

NOX4, part of the NADPH oxidase family, is a significant contributor to ROS production in the central nervous system (Boonpraman et al. 2023). Growing evidence indicated that NOX4 is crucial in neurodegenerative processes, including PD (Lin et al. 2025; Xing et al. 2023), Alzheimer’s disease (Guan et al. 2022; Maimaiti et al. 2024), and amyotrophic lateral sclerosis (Seredenina et al. 2016), by accelerating oxidative stress, mitochondrial dysfunction, and cell ferroptosis. In models of PD, elevated NOX4 expression has been associated with dopaminergic neuron loss and exacerbation of motor deficits (Lin et al. 2025). Beyond its role in neurodegeneration, NOX4 has also been linked to neuropathic pain (Wack et al. 2021). NOX4-mediated ROS production can activate inflammatory pathways, sensitize nociceptive neurons, and disrupt neuronal-glial communication, thereby promoting the initiation and maintenance of chronic pain (Kallenborn-Gerhardt et al. 2022). Thus, NOX4 acts as a key mediator linking oxidative stress to both neurodegeneration and neuropathic pain. Here, our study first demonstrated that CYLD stabilized NOX4 protein by inhibiting its ubiquitination. Overexpression of NOX4 counteracted the suppressive effect of CYLD silencing on ferroptosis in MPP^+^-triggered SH-SY5Y cells. Besides, YTHDF2 mitigated ferroptosis-associated alterations and pain sensitivity in vivo, and these changes were associated with modulation of the CYLD/NOX4 axis. These results revealed a novel CYLD/NOX4 axis that links protein stability control to ferroptosis and neuronal damage in PD, offering novel mechanistic understanding of the function of deubiquitination in neurodegeneration and pain.

This study has several limitations. First, although YTHDF2 overexpression improved pain-related behaviors and concurrently attenuated ferroptosis-associated changes in MPTP-induced mice, we did not perform pharmacological rescue experiments with ferroptosis inhibitors or iron chelators. Therefore, the relationship between ferroptosis and PD-associated pain in the present study remains correlative rather than strictly causal. In future studies, we will further validate this causal relationship using ferroptosis inhibitors such as ferrostatin-1. Second, part of the mechanistic evidence for the YTHDF2/CYLD/NOX4 axis was obtained from in vitro cell models, which cannot fully reflect the complexity of pain regulation in vivo. Third, because YTHDF2 is a broad m6A reader, other downstream transcripts and signaling pathways may also contribute to the observed phenotypes. Future studies are needed to validate the causal contribution of ferroptosis to PD-related pain and to further define the broader regulatory network downstream of YTHDF2. Besides, CYLD is known to regulate not only ferroptosis-related processes but also apoptosis and necroptosis, both of which are involved in MPTP-induced PD models. However, we did not examine these alternative cell death pathways or perform pathway-specific inhibition experiments in the present study. Therefore, whether ferroptosis acts as a primary driver or occurs secondary to other forms of cell death, and our ferroptosis-centered conclusions should be interpreted with caution. Further, CYLD is a well-established repressor of NF-κB pathway, which plays a critical role in cell survival and inflammation. However, in the present study, we did not evaluate whether NF-κB signaling is altered in our models or assess its potential contribution to neuronal injury. Therefore, we cannot exclude the possibility that CYLD may also influence PD-related pathophysiology through NF-κB-dependent mechanisms. Future studies will be required to systematically investigate the engagement of NF-κB signaling and its interplay with the CYLD/NOX4 axis. Furthermore, although our MeRIP-qPCR and RIP results support that YTHDF2 recognizes m6A-modified CYLD mRNA, we did not map the exact m6A site on CYLD or perform mutation-based validation. Therefore, the current study does not establish site-specific m6A regulation, and this limitation should be considered when interpreting the mechanism. Future studies will focus on m6A site mapping and functional mutagenesis analysis. Although SH-SY5Y cells were differentiated prior to MPP^+^ exposure, they remain a neuroblastoma-derived cell line and cannot fully represent mature dopaminergic neurons. Furthermore, this in vitro model does not capture the complex neuronal circuitry involved in pain regulation. Therefore, the corresponding mechanistic findings should be interpreted cautiously, and further validation in more physiologically relevant models is needed. Although multiple lines of evidence supported ferroptosis in the present study, apoptosis- and necroptosis-related markers were not examined. Thus, other cell death mechanisms may also participate in this process. As PD-related pain involves complex spinal and peripheral mechanisms, the absence of direct evaluation of these pathways in the present study represents a limitation and warrants further investigation in future studies.

In conclusion, our study identified a novel regulatory axis in which reduced YTHDF2 expression impaired the degradation of m6A-modified CYLD mRNA, leading to CYLD upregulation in PD. Elevated CYLD levels, in turn, stabilized NOX4 protein by inhibiting its ubiquitination, thereby promoting neuronal ferroptosis and being associated with neurodegeneration and pain-related behavioral abnormalities. These findings advance knowledge of the molecular events driving PD progression and suggest that targeting the YTHDF2/CYLD/NOX4 pathway may serve as a therapeutic strategy for alleviating neuronal injury and possibly PD-linked pain.

## Supplementary Information

Below is the link to the electronic supplementary material.

Supplementary file1 (PDF 3754 KB)

ESM 2 Supplementary file 2 YTHDF2 was decreased and CYLD was enhanced in MPTP-induced PD mice. Mice were divided into two groups: Sham and MPTP. (A) Body weight changes over time. (B) Locomotor activity assessed by open field test. (C) Motor coordination evaluated by pole-climbing test. (D) Mice were subjected to the rotarod test. (E) Immunohistochemical (IHC) staining of tyrosine hydroxylase (TH) in the striatum. (F) TH protein expression in brain tissues were analyzed by Western blot. (G) The mRNA expression levels of YTHDF2 and CYLD were quantified by qRT-PCR. n = 6. *p < 0.05, **p < 0.01, ***p < 0.001(PNG 1.36 MB)

High Resolution Image (TIF 4306 KB)

ESM 3 Supplementary file3 Supplementary Figure S2. Empty vector and negative control shRNA do not affect basal cellular phenotypes in SH-SY5Y cells. SH-SY5Y cells were divided into Control, oe-NC, and sh-NC groups. (A) Cell viability, (B) intracellular Fe2+ levels, and (C) ferroptosis-related markers (ROS, MDA, and GSH) were assessed. n = 3. ns, not significant(PNG 363 KB)

High Resolution Image (TIF 1301 KB)

ESM 4 Supplementary file4 MPP+ treatment increased m6A modification of CYLD mRNA in SH-SY5Y cells.(PNG 24.6 KB)

High Resolution Image (TIF 171 KB)

ESM 5 Supplementary file5 CYLD decreased K48-linked but not K63-linked ubiquitination of NOX4(PNG 115 KB)

High Resolution Image (TIF 602 KB)

ESM 6 Supplementary file6 Preliminary validation experiments confirmed that this construct effectively increased YTHDF2 expression in vivo(PNG 26.6 KB)

High Resolution Image (TIF 156 KB)

## Data Availability

The datasets used or analyzed during the current study are available from the corresponding author on reasonable request.

## Abbreviations

ACSL4: Acyl-CoA synthetase long-chain family member 4
Co-IP: Co-immunoprecipitation
CCK-8 Assay: Cell Counting Kit-8
CYLD: Cylindromatosis protein
FTL: Ferritin light chain
GPX4: Glutathione peroxidase 4
YTHDF2: YTH Domain-Containing Family Protein 2
MDA: Malondialdehyde
NOX4: NADPH oxidase 4
PD: Parkinson’s disease
PWT: Paw withdrawal threshold
PWL: Paw withdrawal latency
qRT-PCR: Quantitative Reverse Transcription Polymerase Chain Reaction
RIP: RNA immunoprecipitation
ROS: Reactive oxygen species
SLC7A11: Solute carrier family 7 member 11
MPTP: 1-Methyl-4-phenyl-1,2,3,6-tetrahydropyridine
m^6^A: N6-methyladenosine

## Declarations

### Generative AI and AI-assisted technologies in the writing process

During the preparation of this work the authors did not use any AI-assisted technology.
