# Supplementary material for: Inhibition of YTHDF2-mediated CYLD mRNA degradation promotes neuronal ferroptosis and pain in Parkinson's disease through NOX4 deubiquitination
Source: Cell Biol Toxicol. 2026 Jun 6;42(1):87. doi: 10.1007/s10565-026-10204-0 (PMC13346141; doi:10.1007/s10565-026-10204-0)
Supplement: Supplementary file 1 — Supplementary file1 (PDF 3754 KB) [file 10565_2026_10204_MOESM1_ESM.pdf]

查重.docx

by neyosu pefiia

Submission date: 27-Apr-2026 11:01AM (UTC+0800)
Submission ID: 2944867804
File name: 查重.docx (67.56K)
Word count: 5719
Character count: 36081

Inhibition of YTHDF2-mediated CYLD mRNA degradation promotes neuronal ferroptosis and pain in Parkinson's disease through NOX4 deubiquitination

Abstract

Background: Parkinson’s disease (PD) is featured by progressive neurodegeneration linked to iron-dependent ferroptosis, yet the functions of m^6A RNA-binding proteins and deubiquitinating enzymes in this process remain poorly understood. This work investigates the functional interplay between the m^6A reader YTHDF2 and deubiquitinase CYLD in PD-associated ferroptosis and delineates their downstream molecular mechanisms.

Methods: PD models were established using MPTP-treated C57BL/6 mice and MPP^+-exposed SH-SY5Y neuroblastoma cells. Behavioral assessments (open field, rotarod, and pole tests) and pain sensitivity assays (mechanical allodynia, thermal hyperalgesia) were performed. Molecular analyses included qRT-PCR, Western blot, RNA immunoprecipitation (RIP), co-immunoprecipitation (Co-IP), and ubiquitination assays. Ferroptosis markers (Fe^{2+}, ROS, MDA, and GSH) and key regulators (ACSL4, GPX4, SLC7A11, and FTL) were quantified. Gain- and loss-of-function experiments for YTHDF2, CYLD, and NOX4 were conducted to validate regulatory relationships.

Results: MPTP/MPP^+ treatment downregulated YTHDF2 and upregulated CYLD, exacerbating ferroptosis, as evidenced by mitochondrial damage, elevated Fe^{2+}/ROS/MDA, reduced GSH, and altered level of ferroptosis-associated proteins (ACSL4 increased, GPX4/SLC7A11/FTL decreased). YTHDF2 overexpression suppressed ferroptosis, at least in part, by recognizing m6A-modified CYLD mRNA and promoting its degradation. Moreover, CYLD stabilized NOX4 by inhibiting its ubiquitination. Rescue experiments confirmed that CYLD depletion attenuated ferroptosis, an effect that was rescued by NOX4 overexpression. In MPTP-induced mice, YTHDF2 overexpression alleviated motor deficits (improved locomotion, rotarod performance), and reduced pain hypersensitivity, while mitigating ferroptosis markers and nigral mitochondrial pathology.

Conclusion: Our study uncovered the YTHDF2/CYLD/NOX4 axis as a novel ferroptosis regulator in PD, revealing a dual epitranscriptomic-posttranslational therapeutic target for neuroprotection.

Keywords: YTHDF2; CYLD; NOX4; Parkinson’s disease; Ubiquitination; m^6A

Introduction

Parkinson's disease (PD) is a progressive neurodegenerative disease caused by dopaminergic neuronal loss in the substantia nigra and is characterized by motor symptoms such as bradykinesia, tremor, rigidity, and postural instability[1, 2]. Beyond motor dysfunction, various non-motor manifestations—including cognitive deterioration, mood disturbances, sleep disorders, and pain—substantially contribute to the disease burden[3, 4]. Among these, pain is reported in up to 85% of PD patients and has been widely regarded as a significant factor impairing quality of life[5]. Pain in PD presents as musculoskeletal, radicular-neuropathic, dystonia-induced, akathisia-associated, and centrally-originated forms[6, 7]. However, the management of PD-associated pain remains challenging, as current therapeutic options are often insufficient or only partially effective. Although alterations in dopaminergic pathways have been implicated[8], the pathophysiological mechanisms underlying PD pain are highly complex and not yet fully elucidated. A deeper understanding of the molecular and cellular mechanisms responsible for PD-related pain is urgently needed to discover innovative treatment targets and enhance patient outcomes for affected individuals.

Ferroptosis is a distinct form of regulated cell death marked by iron dependency and the buildup of lipid peroxidation products, fundamentally differing from apoptosis, necrosis, and other cell death pathways[9, 10]. Emerging studies demonstrated that ferroptosis significantly contributes to PD pathogenesis, mainly via iron accumulation and oxidative damage in dopaminergic neurons[11]. Recent studies have shown that NOX4, a principal source of ROS, exacerbates PD pathology by promoting neuronal ferroptosis and amplifying neuroinflammation[12]. Furthermore, NOX4 has been linked to neuropathic pain[13-15], suggesting its potential involvement in pain processing pathways. However, the function of NOX4 in PD remains insufficiently characterized, and the upstream regulatory mechanisms governing NOX4 activation are still poorly understood. In particular, the specific contribution of NOX4 to PD-associated pain has not yet been elucidated, warranting further investigation to clarify its mechanistic role and therapeutic potential.

CYLD plays a principal function in modulating multiple biological processes, including immune responses, cell proliferation, and apoptosis[16, 17]. Previous studies have demonstrated that CYLD level is augmented under conditions of cellular stress, and its activity has been shown to impact the survival of neurons by modulating signaling pathways involved in inflammation and oxidative stress[18]. For instance, in models of neurodegeneration, CYLD inhibition has been linked to a reduction in oxidative damage and a delay in neuronal cell death, suggesting a protective role against neurodegenerative diseases[19]. Specifically, CYLD has been shown to lower PARIS levels, a key factor that induces mitochondrial dysfunction and impairs neuronal survival in PD[20, 21]. By inhibiting CYLD, PARIS expression is reduced, leading to improved mitochondrial function and enhanced bioenergetics, which may contribute to neuronal resilience. However, the specific mechanisms by which CYLD influences PD pathology remain unclear, and further investigation is required. Through database mining and bioinformatic analyses, we identified that NOX4 undergoes ubiquitination

modification and may interact with CYLD. Given CYLD's function as a deubiquitinase, we hypothesize that CYLD may stabilize NOX4 by removing its ubiquitin chains, thereby promoting NOX4 accumulation. Elevated NOX4 levels could, in turn, enhance neuronal ferroptosis and neuroinflammation, contributing to PD development and the progression of PD-associated neuropathic pain. Thus, investigating the CYLD/NOX4 axis may provide new perspectives on PD pathogenesis and identify potential therapeutic targets.

Recent studies have emphasized the importance of N6-methyladenosine (m^6A) RNA modification in the development and progression of PD[22, 23]. m^6A modification, which regulates mRNA stability, splicing, and translation, is mediated by a dynamic balance between "writers" (such as METTL3 and METTL14), "erasers" (such as FTO), and "readers" (such as YTHDF2)[24, 25]. In PD patients, the expression levels of METTL3 and METTL14[23] are significantly reduced, whereas FTO[26] expression is elevated, suggesting a global disruption of m^6A homeostasis. Although the contribution of m^6A dysregulation to PD pathogenesis is increasingly recognized, the specific molecular mechanisms involving YTHDF2 in PD remain largely unknown. Our preliminary analysis identified multiple putative m^6A modification sites within the CYLD mRNA. Given that YTHDF2 accelerates mRNA degradation by recognizing m^6A-modified transcripts, we hypothesize that reduced YTHDF2 levels in PD impair the degradation of CYLD mRNA, resulting in its accumulation. These findings suggested a novel epitranscriptomic regulatory pathway contributing to dopaminergic neuron degeneration and pain in PD.

This study aimed to investigate whether YTHDF2 regulates CYLD mRNA stability by recognizing m6A-modified CYLD transcripts, thereby modulating the CYLD/NOX4 axis and influencing ferroptosis-associated changes and pain-related behaviors in PD models.

Materials and Methods

Experimental animals

Twenty-four male C57BL/6 mice (8-10 weeks old, weighing 20-22 g) were obtained from the Hunan SJA Laboratory Animal Co., Ltd. (Changsha, China). The animals were housed under specific pathogen-free conditions at a controlled temperature of 25 ± 2°C with 60-70% relative humidity maintained on a 12-h light/dark cycle. Throughout the experiment, mice received food and water ad libitum. After acclimatization for 1 week, animals were randomly allocated into four groups (n=6/group): sham control, MPTP model, MPTP + AAV-oe-NC, and MPTP + AAV-oe-YTHDF2.

Mice received daily intraperitoneal administration of MPTP at 25 mg/kg for 7 consecutive days to induce PD-like pathological changes. Mice in the sham group received equal volumes of normal saline. The adeno-associated virus vectors (AAV-oe-YTHDF2 and the corresponding negative control, AAV-oe-NC) were synthesized by Hanheng Biotechnology (Shanghai) Co., Ltd. Two days prior to MPTP administration, mice in the MPTP + AAV-oe-NC and MPTP + AAV-oe-YTHDF2 groups were subjected to stereotactic injection of AAV-oe-NC or AAV-oe-YTHDF2 into both sides of the

substantia nigra. For in vivo overexpression, an AAV5 vector driven by the mouse neuron-specific Mecp2 promoter was used. Preliminary validation experiments confirmed that this construct effectively increased YTHDF2 expression in vivo. All mice were subjected to behavioral tests 5 days after the last injection.

Behavioral tests

All behavioral experiments were carried out and analyzed by researchers who had no access to the grouping information.

The rotarod and pole tests are commonly employed for assessing motor function and movement slowness in neurological disease models[27].

Rotarod test: Mice were first trained to stay on the rotarod at a constant speed of 4 revolutions per minute (r/min) until they could maintain balance for more than 2 minutes. During testing, the rotarod was accelerated from 4 to 40 rpm within 5 min. Fall latency was recorded for each mouse, and the test was repeated three times per animal.

Pole test: A 50-cm-high wooden pole (1 cm in diameter) with a rough surface was vertically positioned in the home cage. During training, mice were placed on top of the pole and allowed to descend to the cage bottom three times. For testing, mice were placed facing downward on the pole top, and the descent time was recorded. Three trials were performed for each animal.

Open field test

Each mouse was individually placed at the center of an open field arena (40 × 40 × 40 cm) for a 10-minutes observation period. A video tracking system (Shanghai, China) was used to monitor and analyze locomotor activity and exploratory behavior. After each session, the chamber was cleaned with 75% ethanol to eliminate odor cues. Parameters including movement trajectory, total distance traveled, average speed, and distance covered in the central area were recorded for analysis.

Immunohistochemistry (IHC)

Mouse brains were transcardially rinsed with ice-cold saline, fixed with 4% paraformaldehyde, and cryoprotected in 30% sucrose before being cut into 25-μm sections on a cryostat. Endogenous peroxidase was blocked with 3% hydrogen peroxide for 10 min, and sections were then washed with PBS and blocked using 1% BSA containing 0.3% Triton X-100. Subsequently, sections were incubated overnight at 4°C with anti-TH rabbit monoclonal antibody (1:200, #58844, CST), followed by HRP-conjugated secondary antibody. Signals were developed with a DAB kit (Servicebio), and images were acquired after scanning.

Immunofluorescence Staining

Mouse brains were fixed in 4% paraformaldehyde, dehydrated in 30% sucrose, and sectioned at 25 μm. Sections were blocked with 5% BSA and 0.3% Triton X-100 for 1

hour. Primary antibodies against YTHDF2 (1/50, ab246514, Abcam) and NeuN (1/100, ab177487, Abcam) were applied overnight at 4°C. After washing, sections were incubated with fluorophore-conjugated secondary antibodies for 1 h, and nuclei were stained with DAPI. Images were captured using a fluorescence microscope.

Mechanical allodynia

Paw withdrawal threshold (PWT) was assessed using Stoelting's von Frey filaments. Mice were acclimated for 30 minutes in Plexiglass chambers prior to testing. Mechanical stimuli were delivered to the plantar region of the ipsilateral hind paw using von Frey filaments ranging from 0.02 to 4.0 g, and the response threshold was determined with the up-and-down paradigm. A brisk paw withdrawal or licking directed toward the stimulated area was considered a positive nociceptive response. PWT was determined from the response sequence and the force of the final applied filament. Data were log-transformed for statistical analysis.

Thermal hyperalgesia

Thermal sensitivity was measured using the Hargreaves method. Mice were first acclimated for 30 minutes in Plexiglass enclosures. An infrared radiometer was then used to determine paw withdrawal latency (PWL) of the ipsilateral hind paw in response to a thermal stimulus. A 30-s cut-off time was applied to prevent thermal injury.

Electron Microscopy

To observe ultrastructural changes, freshly prepared 1 mm-thick SN/STR tissue slices or cell pellets from cultured cells were fixed in 2.5% glutaraldehyde overnight at 4 °C. Samples were then washed three times with 0.1 M PBS and post-fixed in 1% osmium tetroxide for 2 h at 4 °C. After dehydration through a graded ethanol series, samples were embedded in epoxy resin. Randomly selected ultrathin sections were double-stained with uranyl acetate and lead citrate, followed by observation under a TEM (Hitachi).

Cell culture

HEK293T and SH-SY5Y cell lines were sourced from the Chinese Academy of Sciences Cell Bank and grown in DMEM (VivaCell) containing 10% FBS and 1% penicillin-streptomycin under standard conditions at 37 °C with 5% CO_2. SH-SY5Y cells were cultured under the same conditions and differentiated before subsequent experiments. Briefly, SH-SY5Y cells (passage number ≤ 4) were differentiated by treatment with 1 µM all-trans retinoic acid (RA) for 5 days in complete culture medium containing 1% FBS. After differentiation, the cells were used for subsequent experiments. To establish a PD cellular model, SH-SY5Y were challenged with 1 mM MPP^+ for 24 h.

The overexpression plasmids for pcDNA3.1-YTHDF2 (oe-YTHDF2), pcDNA3.1-CYLD (oe-CYLD), and pcDNA3.1-NOX4 (oe-NOX4), along with pcDNA3.1 plasmid (oe-NC), as well as short hairpin (sh) RNA targeting YTHDF2 (sh-YTHDF2#1, sh-YTHDF2#2, and sh-YTHDF2#3), CYLD (sh-CYLD), and a negative control (sh-NC),

were all purchased from GenePharma (Shanghai, China). Following plasmid or shRNA delivery via Lipofectamine 2000 (Invitrogen), cells were cultured for 48 h.

CCK-8 Assay

SH-SY5Y cells were dispensed into 96-well plates at 2 × 10^3 cells/well and maintained for 24 h. After another 24 h of incubation, cell viability was assessed by recording the absorbance at 450 nm with a microplate reader.

Live/Dead Staining

Treated cells were stained with Calcein-AM/PI (Beyotime) for 30 min and imaged by fluorescence microscopy. Viable cells (green) and dead cells (red) were quantified from three random fields.

Western blot assay

Total protein was extracted from cells and tissues using RIPA buffer and quantified with a BCA kit (Beyotime, China). Protein samples (30 μg) were electrophoretically separated and transferred to PVDF membranes. Membranes were blocked in 5% non-fat milk and incubated overnight at 4°C with TH (#58844, 1:1000, Cell Signaling Technology), YTHDF2 (ab220163, 1:1000, Abcam), ACSL4 (#38493, 1:1000, Cell Signaling Technology), GPX4 (ab125066, 1:1000, Abcam), FTL (SC0620, 1:5000, Thermo Fisher Scientific, TFS), SLC7A11 (PA1-16893, 1:1000, TFS), CYLD (#43-7700, 1:1000, TFS), NOX4 (MA5-32090, 1:2000, TFS), and β-actin (MA1-140, 1:5000, TFS)-specific antibodies. Following washing, membranes were incubated with secondary antibodies, and immunoreactive bands were detected using an ECL kit. Band intensities were quantified with ImageJ software.

qRT-PCR

TRIzol Reagent (Invitrogen) was used to obtain total RNA, which was subsequently converted into cDNA using the High-Capacity cDNA Reverse Transcription Kit (Applied Biosystems). Gene expression was quantified on a LightCycler 480 II system (Roche) with ChamQ Universal SYBR qPCR Master Mix (Vazyme), using GAPDH as the internal control and the $2^{-\Delta\Delta Ct}$ method for analysis. Table 1 presents the primer sequences.

Table 1. The primers used in work

Gene	Forward (5’-3’)	Reverse (5’-3’)
YTHDF2(h)	TAGCCAGCTACAAGCACACCAC	CAACCGTTGCTGCAGTCTGTGT
CYLD(h)	TCAGGCTTATGGAGCCAAGAA	ACTTCCCTTCGGTACTTTAAGGA
NOX4(h)	TCTGGCTCT-CCATGAATGTC	CTGCTTGGAACCTTCTGTGA
GAPDH(h)	CTGACTTCAACAGCGACACC	GTGGTCCAGGGGTCTTACTC
YTHDF2(m)	GGTTCTGTGCATCAAAAGGATGG	CCAAAGAATAGGAA AAGCCAATGG
CYLD(m)	GGATGACTCTGCCTGGCTTTTC	CAGGTCCTCCAGAGACATCTTC

GAPDH(m)	AGCCCAAGATGCCCTTCAGT	CCGTGTTCCTACCCCCAATG

Intracellular Fe^{2+} content

After washing, cells were incubated with the FerroOrange probe (#F374, Dojindo, Japan) diluted in serum-free medium to a final concentration of 1 μM. After 30 minutes at 37°C, fluorescent signals were visualized with an inverted microscope system.

Measurement of ROS, MDA, GSH

The levels of ROS, MDA, and GSH were tested using the Reactive Oxygen Species Assay Kit (#S0033, Beyotime), MDA Assay Kit (#S0131S, Beyotime), and GSH Assay Kit (#S0053, Beyotime), respectively.

MeRIP-qPCR

MeRIP-qPCR was conducted using the EpiQuik CUT&RUN m6A RNA Enrichment Kit (Epigentek). Fragmented RNA was enriched with anti-m6A antibody (#202003, Synaptic Systems) or IgG control through protein A/G magnetic beads, followed by cDNA synthesis and RT-qPCR analysis.

RIP assay

RIP assays were conducted in HEK293T cells transfected with sh-YTHDF2 or sh-NC. Lysates were incubated overnight at 4°C with anti-YTHDF2 antibody or IgG control, followed by capture with Protein A/G magnetic beads. The enriched RNAs were purified, reverse-transcribed, and quantified by qPCR for CYLD, with data normalized to Input.

RNA stability assay

RNA stability was examined by treating sh-NC- or sh-YTHDF2-transfected HEK293T cells with actinomycin D (5 μg/mL; HY-17559, MedChemExpress). CYLD mRNA levels were then quantified by RT-qPCR at the indicated time points.

Protein stability analysis

Protein degradation was examined in oe-CYLD- or oe-NC-transfected 293T cells after CHX exposure (10 μg/mL; Sigma-Aldrich) for 0–8 h. Protein abundance was then assessed by Western blot.

Co-IP analyses

HEK293T cells were used for the ubiquitination-related assays because of their high transfection efficiency, which makes them particularly suitable for plasmid-based protein expression and interaction studies. Cell lysates were prepared in Co-IP buffer and cleared by centrifugation at 13,000 × g for 10 min at 4°C, followed by overnight

incubation with anti-CYLD antibody (#11110-1-AP, Proteintech) or control IgG (#02-6502, Thermo Fisher), and then with Protein A/G PLUS-Agarose beads (#P2012, Beyotime). After washing, the bound proteins were eluted and analyzed by Western blot for NOX4 and CYLD expression. Additionally, an anti-ubiquitin antibody (#3936, Cell Signaling Technology) was used to evaluate NOX4 ubiquitination levels.

Statistical analyses

Statistical analyses were performed with SPSS 25.0 and GraphPad Prism 7.0. Differences between two groups were assessed by two-tailed t-tests, and multiple group comparisons by one-way ANOVA with Tukey's post hoc test. Data are shown as mean ± SEM. A p-value < 0.05 was considered significant.

Results

YTHDF2 was decreased and CYLD was enhanced in MPTP-induced PD mice

A murine model of MPTP-stimulated PD was established to probe the role of YTHDF2 and CYLD in PD. Mouse body weight was recorded daily at a fixed time during the experiment. The MPTP group had lower body weight relative to the sham group, though no statistically significant difference was observed (**Figure S1A**). MPTP-stimulated mice exhibited a distinct suppression in total movement distance, decreased average speed, and prolonged immobility time in the open field test (**Figure S1B**). Besides, MPTP-triggered mice exhibited impaired pole-climbing ability and significantly reduced rotarod retention time (**Figure S1C and D**). We measured the TH expression in striatum and brain tissues and found that it was decreased in PD mice compared with sham mice (**Figure S1E and F**). Further, MPTP-induced PD mice exhibited distinct depletion of YTHDF2 and upregulation of CYLD in the striatum (**Figure S1G**). These findings suggest that downregulation of YTHDF2 and upregulation of CYLD may underlie the development of MPTP-induced PD.

YTHDF2 was downregulated and CYLD was upregulated in MPP^+-triggered SH-SY5Y cells

To mimic PD in vitro, SH-SY5Y cells were challenged with 1 mM MPP^+ for 24 h. As unveiled in **Figure 1A**, YTHDF2 expression was downregulated, while CYLD level was upregulated in MPP^+-exposed cells. In addition, cell viability was significantly reduced following MPP^+ exposure (**Figure 1B**). To further evaluate cell damage, Calcein-AM/PI co-staining was utilized. As expounded in **Figure 1C**, MPP^+ treatment increased the number of dead cells and diminished the number of live cells. Moreover, ferroptosis was observed in MPP^+-exposed SH-SY5Y cells, as evidenced by mitochondrial fragmentation, swelling, vacuolization, and loss of mitochondrial structural integrity (**Figure 1D**). These findings collectively confirm the successful establishment of a PD-like cellular model and suggest that MPP^+ induces ferroptosis in SH-SY5Y cells.

Overexpression of YTHDF2 suppressed ferroptosis

YTHDF2 levels were decreased in MPP^+-triggered SH-SY5Y cells, an effect that was

reversed by YTHDF2 overexpression (**Figure 2A and B**). As depicted in **Figure 2C**, YTHDF2 addition reversed the MPP^+-caused diminution of cell viability. Moreover, MPP^+ treatment increased the number of dead cells and diminished the number of live cells, effects that were reversed by YTHDF2 overexpression (**Figure 2D**). Moreover, MPP^+ treatment significantly elevated Fe^{2+} (**Figure 2E**), ROS (**Figure 2F**), and MDA (**Figure 2F**) levels, while reducing GSH (**Figure 2F**) levels in SH-SY5Y cells; these effects were significantly attenuated by YTHDF2 overexpression. Besides, MPP^+ treatment upregulated ACSL4 expression while downregulating GPX4, FTL, and SLC7A11 levels in SH-SY5Y cells. These alterations were partially reversed by YTHDF2 overexpression (**Figure 2G**). These findings indicate that overexpression of YTHDF2 inhibits MPP^+-induced ferroptosis in vitro. Cell viability was comparable among the Control, oe-NC, and sh-NC groups (**Figure S2A**), intracellular Fe^{2+} accumulation (**Figure S2B**), ROS levels, MDA content, or GSH levels (**Figure S2C**) under basal conditions, indicating that the empty vector and negative control shRNA did not affect cellular phenotypes.

YTHDF2 promoted the degradation of CYLD mRNA

Three YTHDF2-targeting shRNAs (shRNA#1, #2, and #3) effectively knocked down YTHDF2 expression, with sh#2 showing the strongest silencing effect and thus being used in later experiments (**Figure 3A and B**). As an m^6A-binding protein, YTHDF2 is known to accelerate mRNA decay by recognizing m^6A-modified transcripts and promoting transcript degradation[28, 29]. Consistent with its role in mRNA destabilization, knockdown of YTHDF2 increased CYLD levels (**Figure 3C and D**). Moreover, MeRIP-qPCR showed increased enrichment of m6A-modified CYLD mRNA in the YTHDF2-silenced group (**Figure 3E**). To further verify the direct effect of YTHDF2 on CYLD mRNA, RIP assay demonstrated that YTHDF2 directly interacts with CYLD mRNA, and its knockdown markedly decreased the enrichment level of CYLD mRNA in the immunoprecipitates (**Figure 3F**). Depletion of YTHDF2 significantly attenuated CYLD mRNA degradation, thereby increasing its transcript stability (**Figure 3G**). These data indicated that YTHDF2 regulates CYLD expression by recognizing m6A-modified CYLD mRNA and promoting its degradation, thereby contributing to post-transcriptional control of CYLD expression.

To further validate whether CYLD mRNA undergoes m6A modification under PD-like conditions, we performed MeRIP-qPCR analysis in SH-SY5Y cells following MPP^+ exposure. As depicted in **Figure S3**, MPP^+ treatment visibly increased the m6A enrichment level of CYLD mRNA compared with the control group, further supporting that CYLD is an m6A-modified transcript in the MPP^+-treated PD cellular model.

YTHDF2 suppressed ferroptosis via regulating CYLD

To investigate whether YTHDF2 mediates ferroptosis in cells through CYLD, SH-SY5Y cells were assigned to the following groups: Control, MPP^+, MPP^++oe-YTHDF2, and MPP^++oe-YTHDF2+oe-CYLD. In MPP^+-exposed SH-SY5Y cells, YTHDF2 downregulated CYLD expression, an effect that was reversed upon CYLD overexpression (**Figure 4A and B**). MPP^+ treatment decreased cell viability, which was

partially rescued by YTHDF2 overexpression; however, this rescue effect was reversed by co-overexpression of CYLD (**Figure 4C**). Besides, MPP^+ led to an augmentation in dead cells and a decline in viable cells. Addition of YTHDF2 partially restored cell proliferation, while co-overexpression of CYLD reversed this effect (**Figure 4D**). Moreover, YTHDF2 overexpression decreased the levels of Fe^{2+} (**Figure 4E**), ROS (**Figure 4F**), and MDA (**Figure 4F**), and elevated GSH (**Figure 4F**) levels; however, these changes were reversed upon CYLD overexpression. Further, MPP^+ treatment increased ACSL4 expression but decreased GPX4, FTL, and SLC7A11 levels in SH-SY5Y cells. Addition of YTHDF2 partially reversed these effects, whereas CYLD overexpression abolished the rescue mediated by YTHDF2 (**Figure 4G**). These data displayed that YTHDF2 modulates ferroptosis in MPP+-treated SH-SY5Y cells, at least in part, by recognizing m6A-modified CYLD mRNA and promoting its degradation.

CYLD stabilized NOX4 protein by inhibiting NOX4 ubiquitination

Subsequently, we investigated the underlying mechanism by which the deubiquitinase CYLD regulates neuronal ferroptosis. The UbiBrowser analysis identified the deubiquitinase CYLD as a potential regulator of NOX4 (**Figure 5A**). CYLD was overexpressed or silenced in 293T cells to evaluate its effect on NOX4. Increased CYLD elevated NOX4 protein, but not NOX4 mRNA, while CYLD silencing showed the opposite trend (**Figure 5B and C**). Besides, Co-IP assay results indicated that the endogenous NOX4 protein was coprecipitated by a CYLD-specific antibody (**Figure 5D**). Further, CYLD overexpression stabilized NOX4 protein levels in 293T cells (**Figure 5E**). Notably, CYLD overexpression decreased NOX4 ubiquitination in 293T cells (**Figure 5F**). Notably, to determine which ubiquitin linkage type was affected by CYLD, we further assessed K48- and K63-linked ubiquitination of NOX4. The results indicated that CYLD obviously decreased K48-linked ubiquitination of NOX4, while exerting no obvious effect on K63-linked ubiquitination (**Figure S5**). These data further implied that CYLD stabilizes NOX4 mainly through K48-linked deubiquitination.

CYLD mediated ferroptosis through NOX4

To explore whether CYLD mediates ferroptosis in SH-SY5Y cells through NOX4, cells were assigned to the following groups: Control, MPP^+, MPP^++sh-CYLD, and MPP^++sh-CYLD+oe-NOX4. In MPP^+-stimulated SH-SY5Y cells, CYLD knockdown reduced both CYLD and NOX4 expression levels, whereas NOX4 overexpression specifically restored NOX4 expression (but not CYLD expression) (**Figure 6A**). Besides, MPP^+ treatment decreased cell viability, which was partially rescued by CYLD silencing; however, this rescue effect was abolished by NOX4 overexpression (**Figure 6B**). Additionally, MPP^+ treatment increased the number of dead cells and reduced viable cells, with CYLD silencing partially restoring cell proliferation. Co-overexpression of NOX4 reversed this effect (**Figure 6C**). Furthermore, CYLD silencing in MPP^+-stimulated cells decreased the levels of Fe^{2+} (**Figure 6D**), ROS (**Figure 6E**), and MDA (**Figure 6E**), while increasing GSH levels (**Figure 6E**); these changes were reversed upon NOX4 overexpression. MPP^+ treatment also upregulated ACSL4 level and downregulated GPX4, FTL, and SLC7A11 levels in SH-SY5Y cells.

CYLD silencing partially reversed these effects, whereas NOX4 overexpression abolished the rescue effect induced by CYLD silencing (**Figure 6F**). These results recommended that CYLD silencing suppressed ferroptosis in MPP^+-treated SH-SY5Y cells through regulation of NOX4.

YTHDF2 alleviated neuronal ferroptosis-associated alterations and pain-related behaviors in PD mouse models, accompanied by regulation of the CYLD/NOX4 axis

In MPTP-induced mice, YTHDF2 expression was significantly downregulated, accompanied by upregulation of CYLD and NOX4. Overexpression of YTHDF2 reversed these changes (**Figure 7A**). Meanwhile, YTHDF2 levels in the striatum, reduced by MPTP, were restored following YTHDF2 overexpression (**Figure 7B**). MPTP treatment also led to a marked decline in TH expression, both in the striatum and brain tissue, which was alleviated by YTHDF2 overexpression (**Figure 7C and D**). Behaviorally, MPTP-treated mice exhibited reduced center crossings and average velocity in the open field test, prolonged pole descent time, and shortened rotarod retention time, all of which were improved by YTHDF2 overexpression (**Figure 7E and F**). Pain sensitivity was also altered, as evidenced by decreased PWT and PWL in the MPTP group, effects that were reversed with YTHDF2 overexpression (**Figure 7G**). Morphologically, mitochondrial shrinkage, cristae loss, and outer membrane rupture in the substantia nigra indicated ferroptosis, which was mitigated by YTHDF2 (**Figure 7H**). Biochemically, MPTP increased MDA and Fe^{2+} levels while decreasing GSH levels (**Figure 7I**), and also upregulated ACSL4 while downregulating GPX4, FTL, and SLC7A11 (**Figure 7J**); these ferroptosis-related alterations were partially reversed by YTHDF2 overexpression. Collectively, these findings implied that YTHDF2 alleviates MPTP-induced neurodegeneration and ferroptosis-associated alterations, while improving pain-related behavioral outcomes.

YTHDF2 restrained CYLD/NOX4-driven neuronal ferroptosis and pain in PD

In PD, reduced YTHDF2 impairs the degradation of m6A-modified CYLD mRNA, leading to CYLD accumulation. Elevated CYLD stabilizes NOX4 by reducing its ubiquitination, thereby enhancing neuronal ferroptosis and contributing to pain-related behavioral abnormalities (**Figure 8**).

Discussion

PD is marked by progressive degeneration of dopaminergic neurons and a range of non-motor symptoms, including chronic pain, which severely affect patients' quality of life[30-32]. Although accumulating evidence has highlighted the importance of m^6A RNA modification in PD pathogenesis [33, 34], the specific molecular mechanisms linking m^6A dysregulation to neuronal ferroptosis and neuropathic pain have remained elusive. In this work, we demonstrated that the decrease in YTHDF2 expression in PD impairs CYLD mRNA degradation, leading to elevated CYLD expression. Our findings revealed a novel m6A/YTHDF2/CYLD/NOX4 signaling axis associated with neurodegeneration, ferroptosis-related alterations, and pain-related behaviors in PD

models, providing novel perspectives on the molecular mechanisms underlying disease progression and identifying potential therapeutic targets for intervention.

Increasing evidence links ferroptosis - a lipid peroxidation-driven, iron-reliant cell death pathway - to PD development[35, 36]. Emerging evidence suggested that iron accumulation, GPX4 dysfunction, and excessive lipid peroxidation contribute to dopaminergic neuronal loss in PD models[37]. Studies have shown that ferroptosis inhibitors, such as ferrostatin-1, can alleviate neurodegeneration in PD, further supporting the importance of ferroptosis in PD progression[38]. Consistent with these findings, our study provided compelling evidence that ferroptosis was robustly activated in PD models. This was supported by a marked increase in key ferroptotic markers, including iron accumulation, ROS, and MDA levels, as well as upregulated ACSL4 expression. Conversely, we observed significant downregulation of ferroptosis defense mechanisms, manifested by reduced GSH levels and reduced expression of GPX4, FTL, and SLC7A11. These findings support a central role of ferroptosis in PD-related neuronal injury and identify ferroptosis modulation as a possible therapeutic strategy.

Growing evidence suggests that m6A RNA methylation is closely involved in neuronal function and neurodegenerative processes[39]. Dysregulation of m6A modification and its key regulators, such as METTL3, METTL14, and FTO, has been implicated in PD pathogenesis [40-42]. Among m^6A readers, YTHDF2 is known to selectively bind m^6A-modified transcripts and promote their degradation, thereby fine-tuning gene expression post-transcriptionally[43]. In the nervous system, increasing evidence suggested that YTHDF2 exerts protective effects against neurodegeneration and injury[44]. Existing research has shown that YTHDF2-mediated mRNA degradation is required for neuronal differentiation, axonal regeneration, and synaptic plasticity[45]. For instance, YTHDF2 has been reported to facilitate axon regeneration after nerve injury by selectively degrading negative regulators of axon growth[46]. Furthermore, in models of ischemic stroke, YTHDF2 alleviates neuronal apoptosis and reduces infarct volume through modulating m^6A-dependent transcript stability[47]. Recent research also indicates that YTHDF2 can mitigate inflammatory responses in microglia, thus providing neuroprotection in neuroinflammatory conditions[48]. Collectively, these findings emphasized the beneficial role of YTHDF2 in preserving neuronal integrity and function, suggesting that impairment of YTHDF2-mediated mRNA regulation may contribute to the pathogenesis of various neurological disorders. In the present work, we demonstrated that YTHDF2 level was markedly decreased in PD models, suggesting a potential link between impaired m^6A-dependent mRNA decay and neuronal injury in PD. Addition of YTHDF2 inhibited ferroptosis in MPP^+-triggered SH-SY5Y cells. It is worth noting that YTHDF2 also alleviated ferroptosis-associated alterations and improved pain-related behaviors in MPTP-induced PD mice. Importantly, our data support a model in which YTHDF2 recognizes m^6A-modified CYLD mRNA and facilitates its degradation, thereby suppressing neuronal ferroptosis. Importantly, although YTHDF2 is known to exert broad post-transcriptional regulatory effects, our data support that the CYLD/NOX4 axis represents a major functional pathway underlying the regulatory role of YTHDF2 in PD-related ferroptosis. Given

the broad regulatory nature of m6A modification and YTHDF2-mediated RNA decay, we cannot exclude the possibility that additional ferroptosis-related transcripts may also be involved under PD-like conditions. However, our findings identified CYLD as a necessary downstream effector of YTHDF2 and further demonstrated that CYLD promotes ferroptosis, at least in part, by stabilizing NOX4 protein through deubiquitination. Thus, CYLD served as an important mechanistic hub linking epitranscriptomic regulation to NOX4-dependent oxidative stress in PD.

The involvement of deubiquitinating enzymes in PD pathogenesis has received growing attention, particularly in the context of neuronal vulnerability[49]. CYLD, initially recognized as an anticancer regulator, is increasingly associated with neurodegenerative disease progression[21, 50]. In the central nervous system, CYLD modulates key pathways, including autophagy, NF-κB, and Wnt/β-catenin, which are critical for neuronal survival and homeostasis[21, 51]. CYLD dysregulation may aggravate neuroinflammatory responses, disrupt mitochondrial homeostasis, and amplify oxidative stress, thereby contributing to neurodegenerative pathology[52]. For example, studies have shown that CYLD exacerbates dopaminergic neuron loss in models of PD by promoting mitochondrial dysfunction and inhibiting mitophagy[20]. Moreover, CYLD has been reported to contribute to axonal degeneration and neuroinflammatory responses following traumatic brain injury[21]. These findings suggested that CYLD acts as a detrimental regulator in the nervous system, and its abnormal activation may amplify neuronal vulnerability under pathological conditions. Here, we found that CYLD level was enhanced in animal and cellular PD models. Consistent with the results of previous studies, CYLD aggravated ferroptosis and promoted the progression of PD.

NOX4, part of the NADPH oxidase family, is a significant contributor to ROS production in the central nervous system[53]. Growing evidence indicated that NOX4 is crucial in neurodegenerative processes, including PD[12, 54], Alzheimer's disease[55, 56], and amyotrophic lateral sclerosis[57], by accelerating oxidative stress, mitochondrial dysfunction, and cell ferroptosis. In models of PD, elevated NOX4 expression has been associated with dopaminergic neuron loss and exacerbation of motor deficits[12]. Beyond its role in neurodegeneration, NOX4 has also been linked to neuropathic pain[14]. NOX4-mediated ROS production can activate inflammatory pathways, sensitize nociceptive neurons, and disrupt neuronal-glial communication, thereby promoting the initiation and maintenance of chronic pain[58]. Thus, NOX4 acts as a key mediator linking oxidative stress to both neurodegeneration and neuropathic pain. Here, our study first demonstrated that CYLD stabilized NOX4 protein by inhibiting its ubiquitination. Overexpression of NOX4 counteracted the suppressive effect of CYLD silencing on ferroptosis in MPP^+-triggered SH-SY5Y cells. Besides, YTHDF2 mitigated ferroptosis-associated alterations and pain sensitivity in vivo, and these changes were associated with modulation of the CYLD/NOX4 axis. These results revealed a novel CYLD/NOX4 axis that links protein stability control to ferroptosis and neuronal damage in PD, offering novel mechanistic understanding of the function of deubiquitination in neurodegeneration and pain.

This study has several limitations. First, although YTHDF2 overexpression improved pain-related behaviors and concurrently attenuated ferroptosis-associated changes in

MPTP-induced mice, we did not perform pharmacological rescue experiments with ferroptosis inhibitors or iron chelators. Therefore, the relationship between ferroptosis and PD-associated pain in the present study remains correlative rather than strictly causal. In future studies, we will further validate this causal relationship using ferroptosis inhibitors such as ferrostatin-1. Second, part of the mechanistic evidence for the YTHDF2/CYLD/NOX4 axis was obtained from in vitro cell models, which cannot fully reflect the complexity of pain regulation in vivo. Third, because YTHDF2 is a broad m6A reader, other downstream transcripts and signaling pathways may also contribute to the observed phenotypes. Future studies are needed to validate the causal contribution of ferroptosis to PD-related pain and to further define the broader regulatory network downstream of YTHDF2. Besides, CYLD is known to regulate not only ferroptosis-related processes but also apoptosis and necroptosis, both of which are involved in MPTP-induced PD models. However, we did not examine these alternative cell death pathways or perform pathway-specific inhibition experiments in the present study. Therefore, whether ferroptosis acts as a primary driver or occurs secondary to other forms of cell death, and our ferroptosis-centered conclusions should be interpreted with caution. Further, CYLD is a well-established repressor of NF-κB pathway, which plays a critical role in cell survival and inflammation. However, in the present study, we did not evaluate whether NF-κB signaling is altered in our models or assess its potential contribution to neuronal injury. Therefore, we cannot exclude the possibility that CYLD may also influence PD-related pathophysiology through NF-κB-dependent mechanisms. Future studies will be required to systematically investigate the engagement of NF-κB signaling and its interplay with the CYLD/NOX4 axis. Furthermore, although our MeRIP-qPCR and RIP results support that YTHDF2 recognizes m6A-modified CYLD mRNA, we did not map the exact m6A site on CYLD or perform mutation-based validation. Therefore, the current study does not establish site-specific m6A regulation, and this limitation should be considered when interpreting the mechanism. Future studies will focus on m6A site mapping and functional mutagenesis analysis. Although SH-SY5Y cells were differentiated prior to MPP^+ exposure, they remain a neuroblastoma-derived cell line and cannot fully represent mature dopaminergic neurons. Furthermore, this in vitro model does not capture the complex neuronal circuitry involved in pain regulation. Therefore, the corresponding mechanistic findings should be interpreted cautiously, and further validation in more physiologically relevant models is needed. Although multiple lines of evidence supported ferroptosis in the present study, apoptosis- and necroptosis-related markers were not examined. Thus, other cell death mechanisms may also participate in this process. As PD-related pain involves complex spinal and peripheral mechanisms, the absence of direct evaluation of these pathways in the present study represents a limitation and warrants further investigation in future studies.

In conclusion, our study identified a novel regulatory axis in which reduced YTHDF2 expression impaired the degradation of m6A-modified CYLD mRNA, leading to CYLD upregulation in PD. Elevated CYLD levels, in turn, stabilized NOX4 protein by inhibiting its ubiquitination, thereby promoting neuronal ferroptosis and being

associated with neurodegeneration and pain-related behavioral abnormalities. These findings advance knowledge of the molecular events driving PD progression and suggest that targeting the YTHDF2/CYLD/NOX4 pathway may serve as a therapeutic strategy for alleviating neuronal injury and possibly PD-linked pain.

查重.docx

ORIGINALITY REPORT

14%	11%	12%	4%
SIMILARITY INDEX	INTERNET SOURCES	PUBLICATIONS	STUDENT PAPERS

PRIMARY SOURCES

1	Ning Chen, Zhenwen Zhang, Fangqin Shen, Daming Lu. "METTL5-mediated m6A modification of UBE3C promotes osteosarcoma progression by suppressing ferroptosis via inducing AHNAK ubiquitination", Journal of Molecular Histology, 2025<br>Publication	2%
2	www.ncbi.nlm.nih.gov<br>Internet Source	1%
3	www.mdpi.com<br>Internet Source	1%
4	www.frontiersin.org<br>Internet Source	1%
5	public-pages-files-2025.frontiersin.org<br>Internet Source	1%
6	www.nature.com<br>Internet Source	1%
7	www.xiahepublishing.com<br>Internet Source	1%
8	www.researchsquare.com<br>Internet Source	<1%
9	Gayathri K. Balasuriya, Kota Tamada, Jun Nomura, Carla Cirillo, Toru Takumi. "Prucalopride ameliorates delayed	<1%

gastrointestinal transit and social behaviour in a mouse model of 15q duplication syndrome", Cold Spring Harbor Laboratory, 2025
Publication

10	Le Zhao, Ruifeng Pei, Yiren Ding, Zhan Su, Deqiang Li, Shuo Zhu, Lu Xu, Hongying Zhao, Wuyuan Zhou. "LOXL4 Shuttled by Tumor Cells–derived Extracellular Vesicles Promotes Immune Escape in Hepatocellular Carcinoma by Activating the STAT1/PD-L1 Axis", Journal of Immunotherapy, 2024<br>Publication	<1 %
11	academic.hep.com.cn<br>Internet Source	<1 %
12	cris.maastrichtuniversity.nl<br>Internet Source	<1 %
13	Zhichao Zou, Zhi Liu, Zhanwei Zhang, Xiaojing Li. "TBX21 knockdown attenuates neuroinflammation induced by intracerebral hemorrhage via the SIRT1-WDR5-H3K4me3 axis", Brain Research Bulletin, 2025<br>Publication	<1 %
14	dirros.openscience.si<br>Internet Source	<1 %
15	link.springer.com<br>Internet Source	<1 %
16	pmc.ncbi.nlm.nih.gov<br>Internet Source	<1 %
17	Hongwei Liu, Qianqian Wang, Wanying Lan, Duanya Liu, Jiangang Huang, Jie Yao. "Radiosensitization effect of quinoline-indole-	<1 %

schiff base derivative 10E on non-small cell lung cancer cells in vitro and in tumor xenografts", Investigational New Drugs, 2024
Publication

18 Jiqing Liu, Yina Shao, Dongdong Li, Chenghua Li. "N6-methyladenosine helps Apostichopus japonicus resist Vibrio splendidus infection by targeting coelomocyte autophagy via the AjULK-AjYTHDF/AjEEF-1α axis", Communications Biology, 2023 <1%
Publication

19 www.imrpress.com <1%
Internet Source

20 Submitted to Higher Education Commission Pakistan <1%
Student Paper

21 Submitted to Salisbury University <1%
Student Paper

22 Submitted to University of Macau <1%
Student Paper

23 www.besjournal.com <1%
Internet Source

24 www.dovepress.com <1%
Internet Source

25 Cuiyuan Huang, Yunping Sun, Jingyi Wu, Di Wu, Li Liu, Wenqiang Li, Wei Wang, Jian Yang, Jing Zhang. "Down-regulation of KMT2D mitigates neointimal hyperplasia following carotid artery injury in diabetic rats", Biochemical and Biophysical Research Communications, 2025 <1%
Publication

26 Ziqi Guo, Yuying Huang, Xiaoling Dong, Linyan Shen, Fuguo Yan, Cheng Yang. "Hypoxia-Induced m6A modification via YTHDF2 stabilizes PFKL to fuel MDSC Glycolysis and hepatocellular carcinoma progression", Functional & Integrative Genomics, 2025 <1%
Publication

---

27 Hong-yang Sun, Jin Wu, Rui Wang, Shun Zhang, Hao Xu, Elena Kaznacheyeva, Xiao-jun Lu, Hai-gang Ren, Guang-hui Wang. "Pazopanib alleviates neuroinflammation and protects dopaminergic neurons in LPS-stimulated mouse model by inhibiting MEK4-JNK-AP-1 pathway", Acta Pharmacologica Sinica, 2022 <1%
Publication

---

28 Shilin Miao, Lele Tian, Xiangyan Zhang, Xianqi Zhang, Pei Liu, Jifeng Cai, Yadong Guo, Changquan Zhang. "Non-invasive time inference of repetitive contusion injuries using hyperspectral imaging and machine learning", Spectrochimica Acta Part A: Molecular and Biomolecular Spectroscopy, 2026 <1%
Publication

---

29 Lin Liu, Songqi Yang, Heng Wang. " α-Lipoic acid alleviates ferroptosis in the MPP -induced PC12 cells via activating the PI3K/Akt/Nrf2 pathway ", Cell Biology International, 2020 <1%
Publication

---

30 Tianyu Jiang, Henglong Hu, Yuhang Yuan, Qiyu Jia. "Escitalopram Attenuates Spinal Cord Injury by Suppressing NLRP3 Inflammasome- <1%

Mediated Pyroptosis via PI3K/AKT Pathway Activation", Brain Mechanisms, 2025
Publication

31 Ziqi Guo, Qiuling Huang, Zhenzhen Cui, Cheng Yang, Liu Yang. "Targeting YTHDF2 with pH-responsive siRNA nanoparticles suppresses MYC m6A modification and restores antitumor immunity in hepatocellular carcinoma", Journal of Nanobiotechnology, 2025
Publication
<1%

32 molecular-cancer.biomedcentral.com
Internet Source
<1%

33 peerj.com
Internet Source
<1%

34 www.ecmjournal.org
Internet Source
<1%

35 www.jneurosci.org
Internet Source
<1%

36 Li, Dai, Zhen-Zhen Huang, Yun-Zhi Ling, Jia-You Wei, Yu Cui, Xiang-Zhong Zhang, He-Quan Zhu, and Wen-Jun Xin. "Up-regulation of CX3CL1 via Nuclear Factor-κB–dependent Histone Acetylation Is Involved in Paclitaxel-induced Peripheral Neuropathy :", Anesthesiology, 2014.
Publication
<1%

37 au-east.erc.monash.edu.au
Internet Source
<1%

38 jcancer.org
Internet Source
<1%

storage.freidok.ub.uni-freiburg.de

39	Internet Source	<1%
40	www.science.org<br>Internet Source	<1%
41	Hua Guo, Zhi-Lin Huang, Wei Wang, Shu-Xiao Zhang et al. "iTRAQ-Based Proteomics Suggests Ephb6 as a Potential Regulator of the ERK Pathway in the Prefrontal Cortex of Chronic Social Defeat Stress Model Mice", PROTEOMICS - Clinical Applications, 2017<br>Publication	<1%
42	Jun Qian, Dong Fang, Hong Lu, Yi Cao, Ji Zhang, Rong Ding, Lingchang Li, Jiege Huo. "Tanshinone IIA promotes IL2-mediated SW480 colorectal cancer cell apoptosis by triggering INF2-related mitochondrial fission and activating the Mst1-Hippo pathway", Biomedicine & Pharmacotherapy, 2018<br>Publication	<1%
43	Min Jiang, Jingyi Han, Qun Ma, Xue Chen, Renjie Xu, Qing Wang, Jia Zheng, Weimin Wang, Jun Song, Yefei Huang, Yansu Chen. "Nicotine-derived NNK promotes CRC progression through activating TMUB1/AKT pathway in METTL14/YTHDF2-mediated m6A manner", Journal of Hazardous Materials, 2024<br>Publication	<1%
44	Hai Sun, Yan Sun. "Lidocaine inhibits proliferation and metastasis of lung cancer cell via regulation of miR-539/EGFR axis", Artificial Cells, Nanomedicine, and Biotechnology, 2019<br>Publication	<1%

45	Jie Zhou, Yanping Wang, Guangyan Zhu, Mingzhe Yan et al. "Pilaralisib inhibits the replication of enteroviruses by targeting the PI3K/AKT signaling pathway", Virology Journal, 2025<br>Publication	<1 %

Exclude quotes	On	Exclude matches	Off
Exclude bibliography	On		
