# Supplementary figures and images for: Inhibition of YTHDF2-mediated CYLD mRNA degradation promotes neuronal ferroptosis and pain in Parkinson's disease through NOX4 deubiquitination
Source: Cell Biol Toxicol. 2026 Jun 6;42(1):87. doi: 10.1007/s10565-026-10204-0 (PMC13346141; doi:10.1007/s10565-026-10204-0)

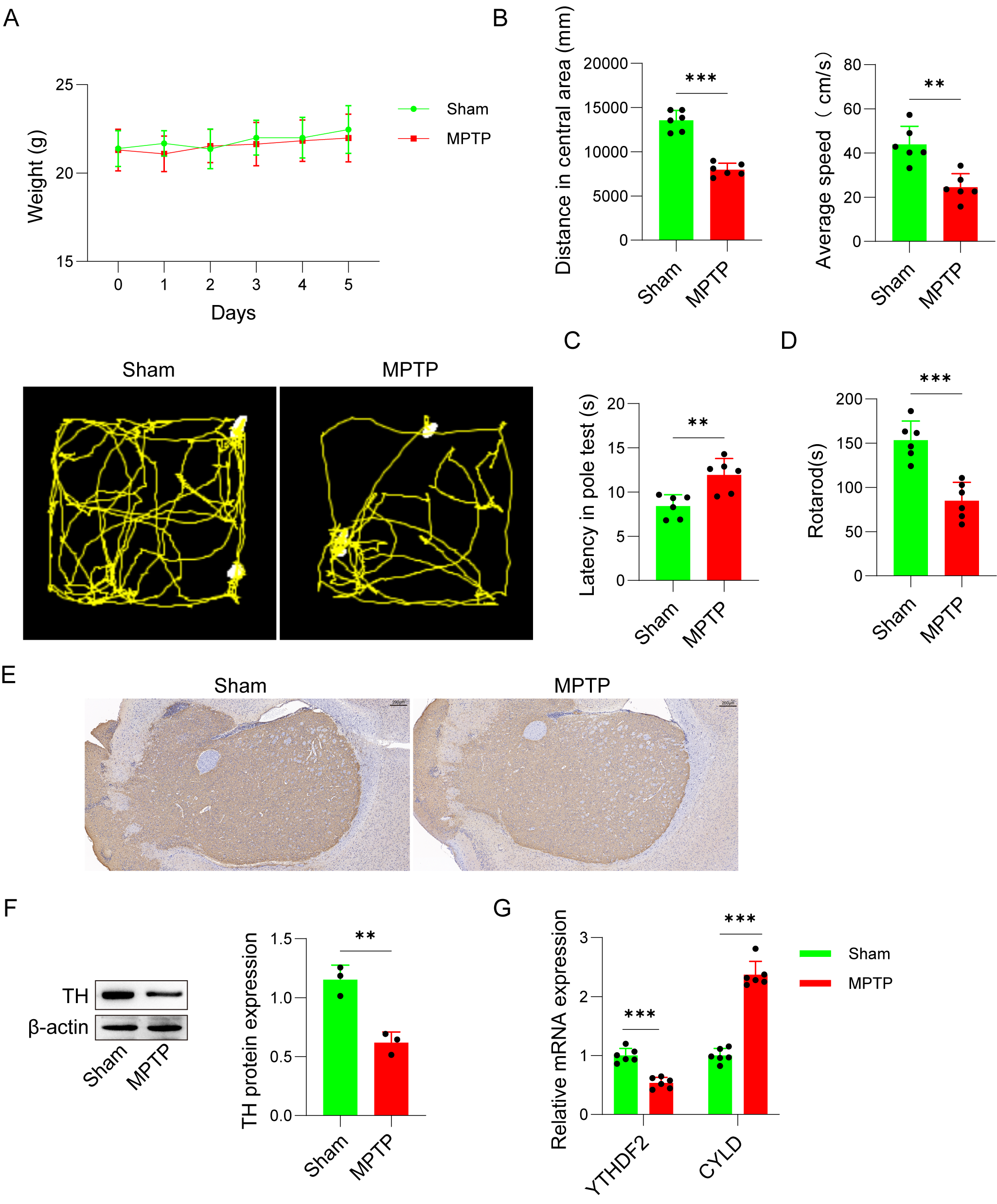

Supplement: Supplementary file 2 — YTHDF2 was decreased and CYLD was enhanced in MPTP-induced PD mice. Mice were divided into two groups: Sham and MPTP. (A) Body weight changes over time. (B) Locomotor activity assessed by open field test. (C) Motor coordination evaluated by pole-climbing test. (D) Mice were subjected to the rotarod test. (E) Immunohistochemical (IHC) staining of tyrosine hydroxylase (TH) in the striatum. (F) TH protein expression in brain tissues were analyzed by Western blot. (G) The mRNA expression levels of YTHDF2 and CYLD were quantified by qRT-PCR. n = 6. *p < 0.05, **p < 0.01, ***p < 0.001(PNG 1.36 MB) [file 10565_2026_10204_Fig8_ESM.png]

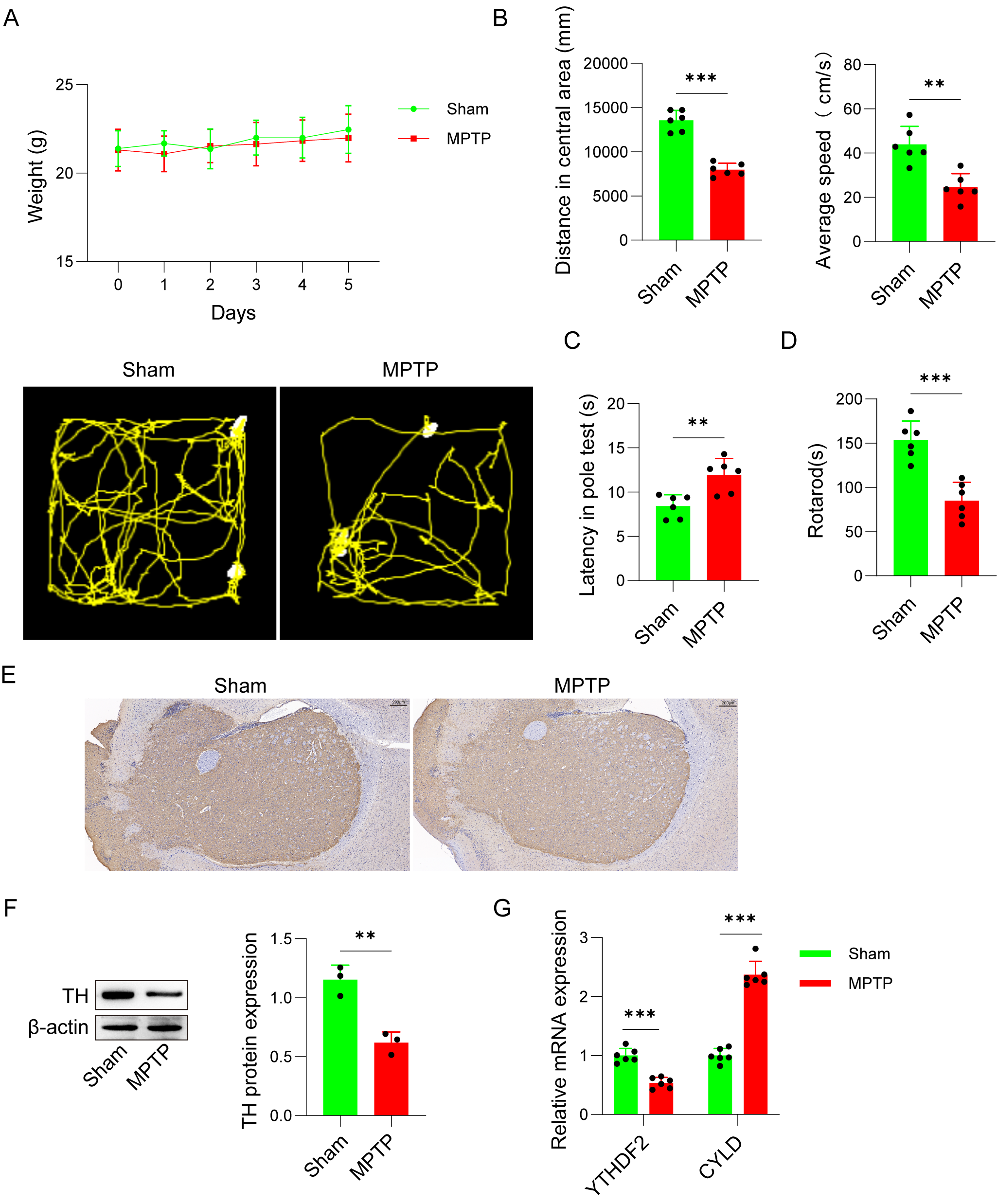

Supplement: Supplementary file 3 — High Resolution Image (TIF 4306 KB) [file 10565_2026_10204_MOESM2_ESM.tif]

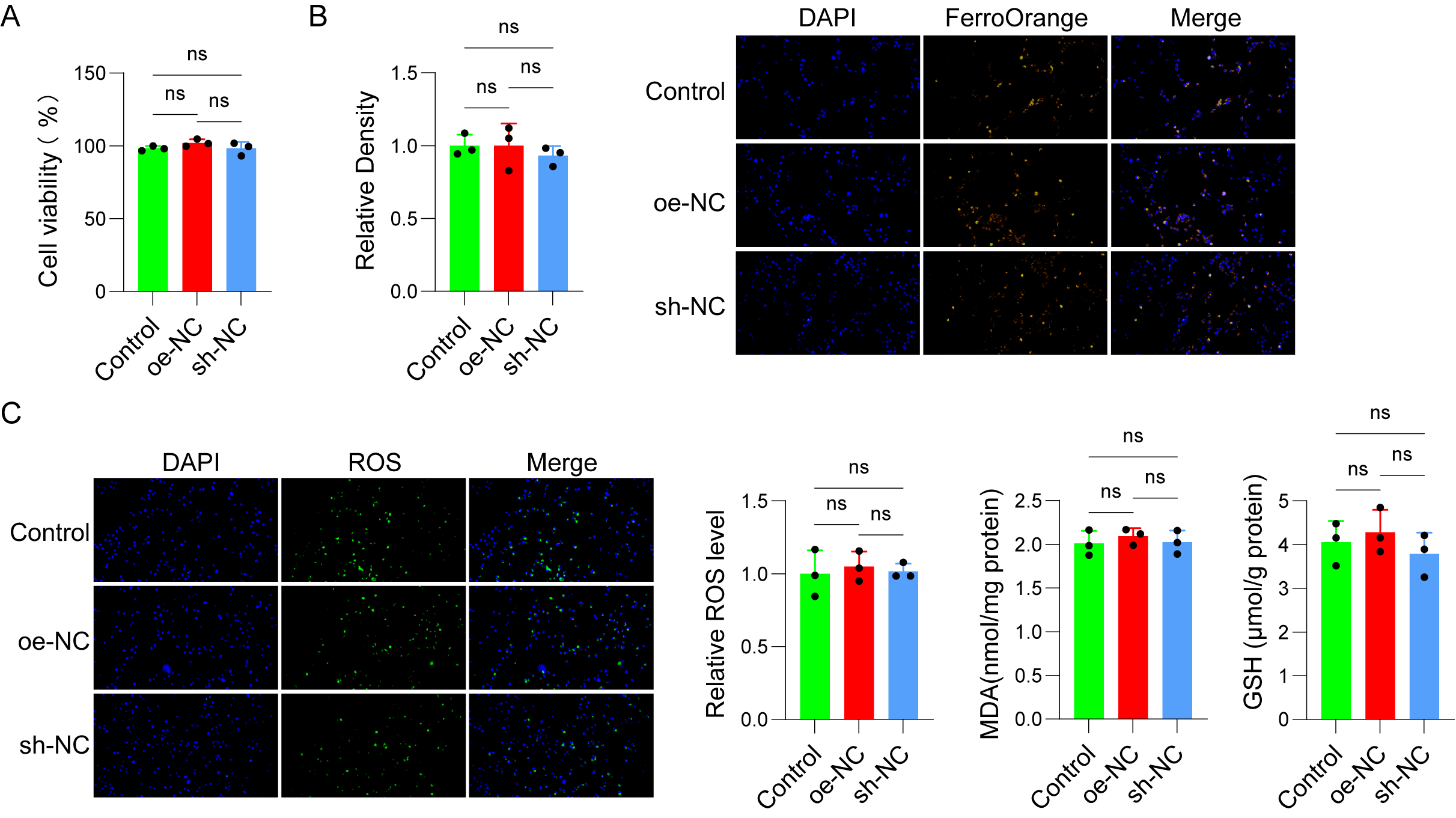

Supplement: Supplementary file 4 — Supplementary file3 Supplementary Figure S2. Empty vector and negative control shRNA do not affect basal cellular phenotypes in SH-SY5Y cells. SH-SY5Y cells were divided into Control, oe-NC, and sh-NC groups. (A) Cell viability, (B) intracellular Fe2+ levels, and (C) ferroptosis-related markers (ROS, MDA, and GSH) were assessed. n = 3. ns, not significant(PNG 363 KB) [file 10565_2026_10204_Fig9_ESM.png]

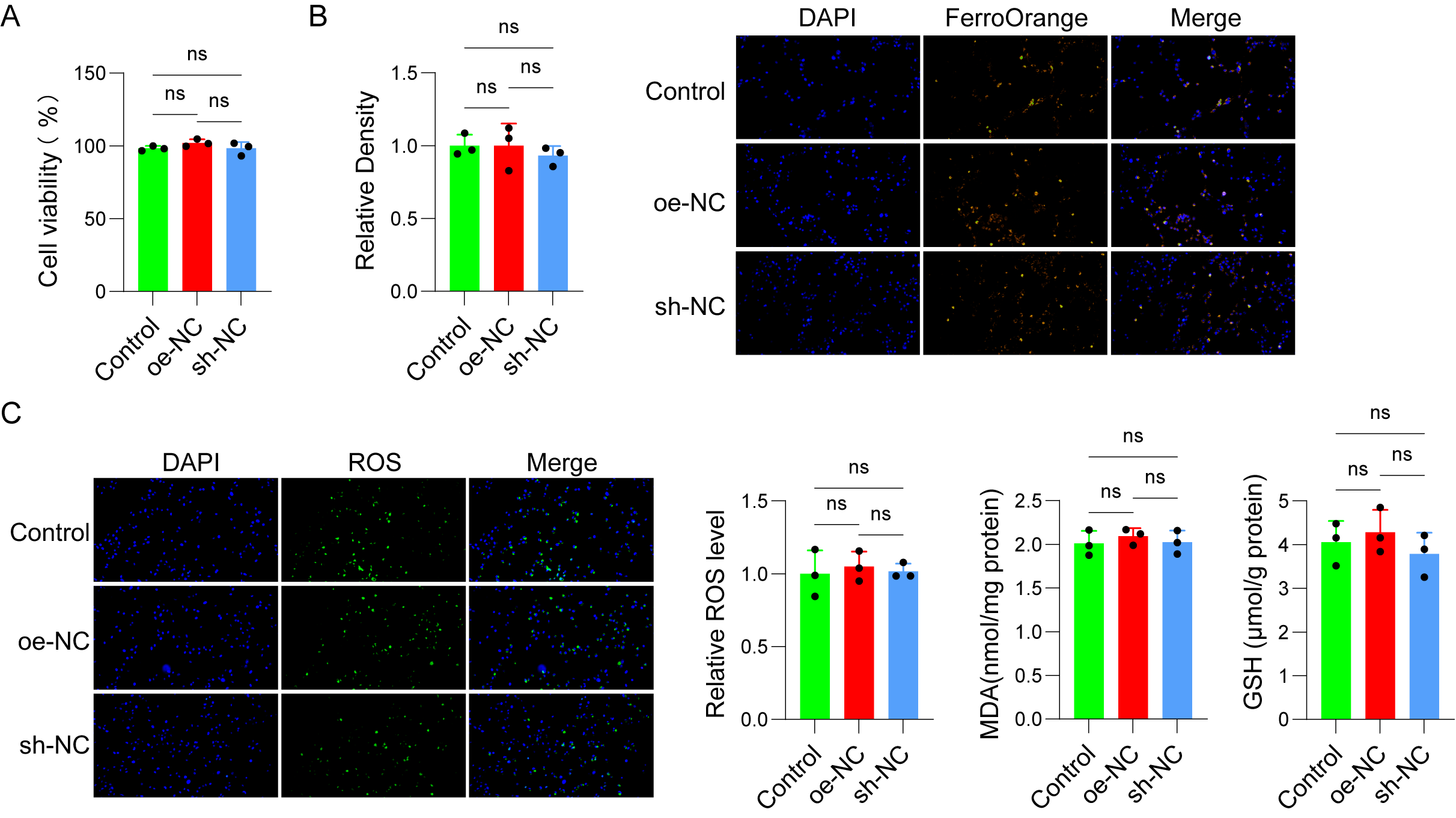

Supplement: Supplementary file 5 — High Resolution Image (TIF 1301 KB) [file 10565_2026_10204_MOESM3_ESM.tif]

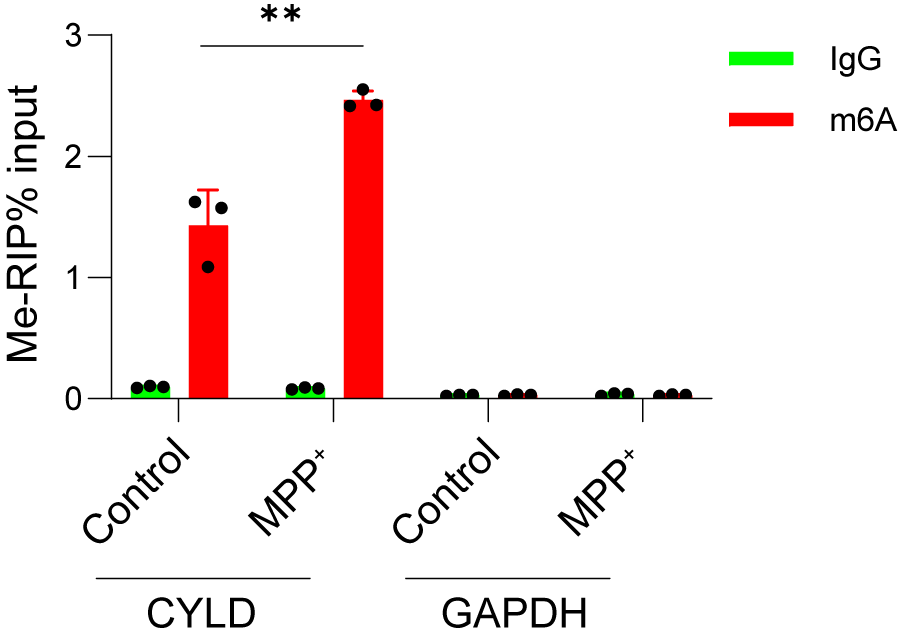

Supplement: Supplementary file 6 — Supplementary file4 MPP+ treatment increased m6A modification of CYLD mRNA in SH-SY5Y cells.(PNG 24.6 KB) [file 10565_2026_10204_Fig10_ESM.png]

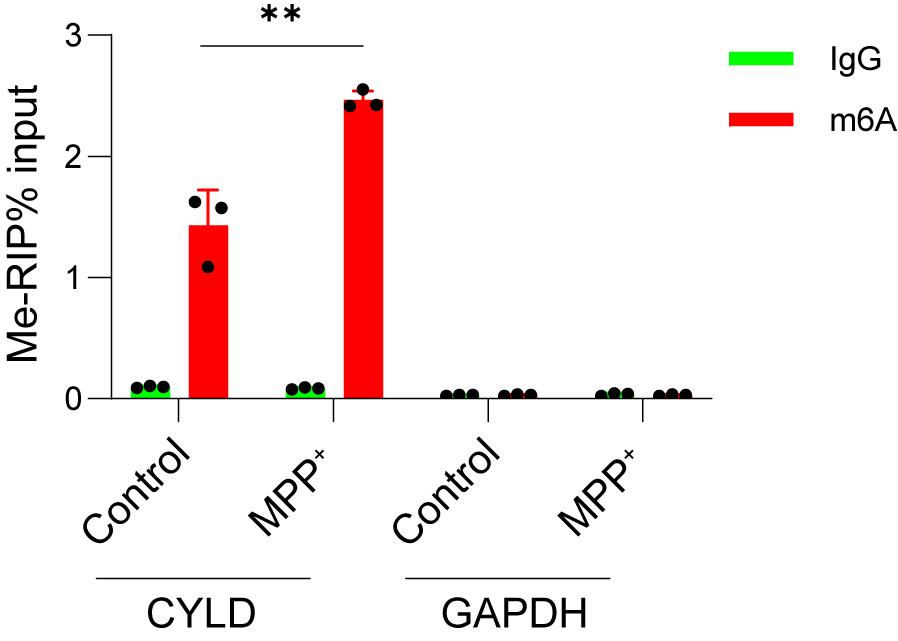

Supplement: Supplementary file 7 — High Resolution Image (TIF 171 KB) [file 10565_2026_10204_MOESM4_ESM.tif]

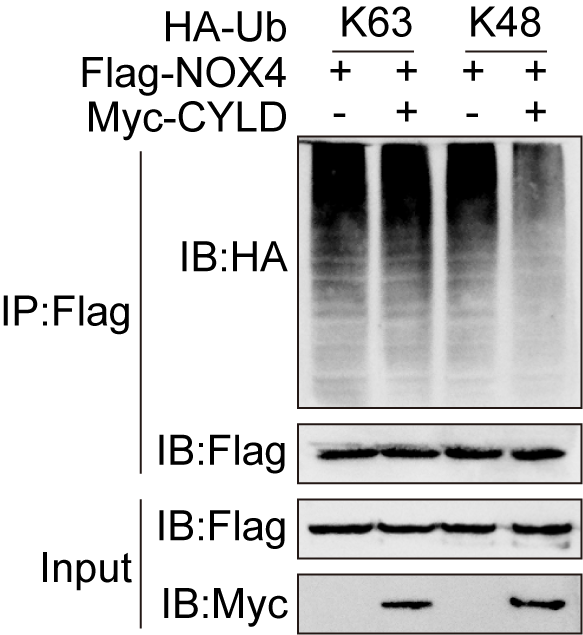

Supplement: Supplementary file 8 — Supplementary file5 CYLD decreased K48-linked but not K63-linked ubiquitination of NOX4(PNG 115 KB) [file 10565_2026_10204_Fig11_ESM.png]

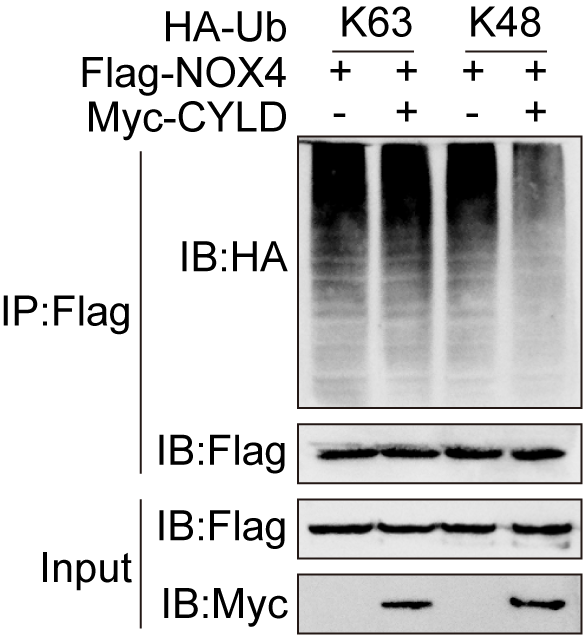

Supplement: Supplementary file 9 — High Resolution Image (TIF 602 KB) [file 10565_2026_10204_MOESM5_ESM.tif]

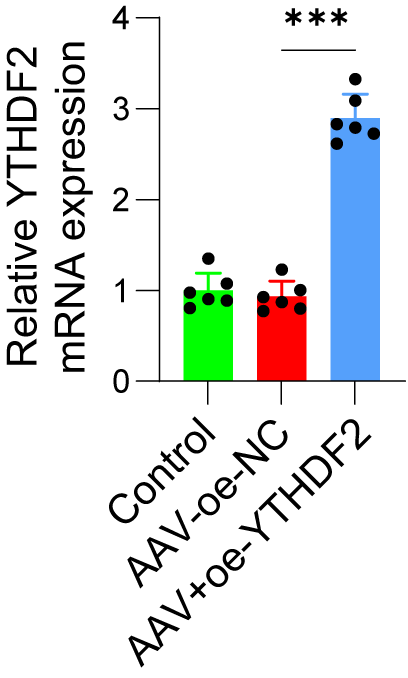

Supplement: Supplementary file 10 — Supplementary file6 Preliminary validation experiments confirmed that this construct effectively increased YTHDF2 expression in vivo(PNG 26.6 KB) [file 10565_2026_10204_Fig12_ESM.png]

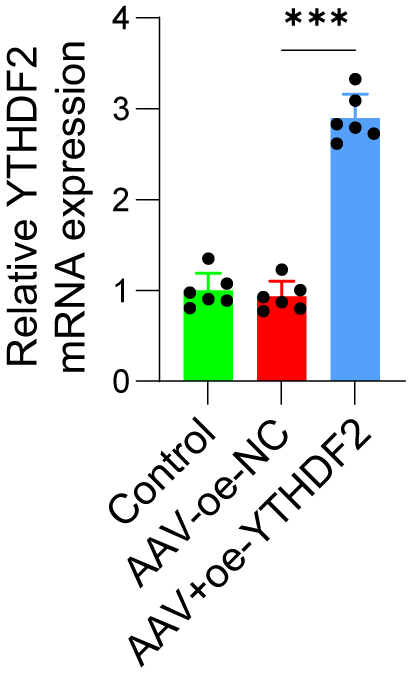

Supplement: Supplementary file 11 — High Resolution Image (TIF 156 KB) [file 10565_2026_10204_MOESM6_ESM.tif]
